# Assessing citation integrity in biomedical publications: corpus annotation and NLP models

**DOI:** 10.1093/bioinformatics/btae420

**Published:** 2024-06-26

**Authors:** Maria Janina Sarol, Shufan Ming, Shruthan Radhakrishna, Jodi Schneider, Halil Kilicoglu

**Affiliations:** Informatics Programs, University of Illinois Urbana-Champaign, Champaign, IL 61820, United States; School of Information Sciences, University of Illinois Urbana-Champaign, Champaign, IL 61820, United States; Department of Computer Science, University of Illinois Urbana-Champaign, Champaign, IL 61801, United States; School of Information Sciences, University of Illinois Urbana-Champaign, Champaign, IL 61820, United States; School of Information Sciences, University of Illinois Urbana-Champaign, Champaign, IL 61820, United States

## Abstract

**Motivation:**

Citations have a fundamental role in scholarly communication and assessment. Citation accuracy and transparency is crucial for the integrity of scientific evidence. In this work, we focus on quotation errors, errors in citation content that can distort the scientific evidence and that are hard to detect for humans. We construct a corpus and propose natural language processing (NLP) methods to identify such errors in biomedical publications.

**Results:**

We manually annotated 100 highly-cited biomedical publications (reference articles) and citations to them. The annotation involved labeling citation context in the citing article, relevant evidence sentences in the reference article, and the accuracy of the citation. A total of 3063 citation instances were annotated (39.18% with accuracy errors). For NLP, we combined a sentence retriever with a fine-tuned claim verification model to label citations as ACCURATE, NOT_ACCURATE, or IRRELEVANT. We also explored few-shot in-context learning with generative large language models. The best performing model—which uses citation sentences as citation context, the BM25 model with MonoT5 reranker for retrieving top-20 sentences, and a fine-tuned MultiVerS model for accuracy label classification—yielded 0.59 micro-F_1_ and 0.52 macro-F_1_ score. GPT-4 in-context learning performed better in identifying accurate citations, but it lagged for erroneous citations (0.65 micro-F_1_, 0.45 macro-F_1_). Citation quotation errors are often subtle, and it is currently challenging for NLP models to identify erroneous citations. With further improvements, the models could serve to improve citation quality and accuracy.

**Availability and implementation:**

We make the corpus and the best-performing NLP model publicly available at https://github.com/ScienceNLP-Lab/Citation-Integrity/.

## 1 Introduction

Citations are foundational to science. It is through citations that scientific claims gain credibility, propagate, and become accepted as facts (Greenberg 2009). Citation-based metrics, such as h-index and journal impact factor, are the primary mechanisms for measuring the scholarly contributions of researchers and journals (Waltman 2016). Due to their central role in scholarly communication and assessment, proper use of citations is a key element of accurate and transparent reporting in a scientific publication and ultimately in its integrity. However, citation inaccuracies and distortions are common (Wager and Middleton 2008, Jergas and Baethge 2015, Pavlovic *et al.* 2021).

Two types of citation errors are often distinguished: *citation accuracy* and *quotation accuracy* errors (Wager and Middleton 2008). The former refers to errors in citation metadata (e.g. author names, publication dates) and the latter to problems in citation content (i.e. whether the statements from the reference papers are accurately reflected in a citing article). Quotation errors are more pernicious in that they are difficult to detect for humans and can distort the integrity of scientific evidence (Greenberg 2009). We characterize such errors as *citation integrity* errors, to clarify their implications. They are the focus of this study.

A growing body of citation analysis studies, mostly targeting various biomedical subdomains, have aimed to determine the prevalence of citation integrity errors (Davids *et al.* 2010, Awrey *et al.* 2011, Jergas and Baethge 2015, Pavlovic *et al.* 2021). Such errors are often categorized as major and minor; the former referring to errors that seriously misrepresent the claims of the reference paper and the latter referring to inconsistencies or factual errors that are not sufficiently serious to alter the meaning of the source (De Lacey *et al.* 1985). A meta-analysis in 2008 found that quotation accuracy studies reported a median error rate of 20% (Wager and Middleton 2008). A more recent meta-analysis showed that 25.4% of medical articles contained a quotation error, roughly half of them severe errors that distorted the claims from the cited articles (Jergas and Baethge 2015). Greenberg (2009) has demonstrated how speculative claims have become accepted facts in Alzheimer’s disease research through citation distortions. A recent bibliometric analysis suggested that inaccurate citations to a letter published in the New England Journal of Medicine in 1980 (Porter and Jick 1980) may have contributed to the opioid epidemic in the United States (Leung *et al.* 2017). More broadly, retracted articles continue to be cited (Van Der Vet and Nijveen 2016, Suelzer *et al.* 2019, Schneider *et al.* 2020, Hsiao and Schneider 2022), potentially leading to propagation of untrustworthy information.

Citation integrity errors are difficult to detect, because they require the reader, editor, or peer reviewer to be very familiar with the cited article and be able to judge whether the cited information is consistent with the original text. Given that an article on average cites about 45 articles (Dai *et al.* 2021) and the judgment requires expertise, this is a challenging task. In this article, we pose the question of whether natural language processing (NLP) techniques can help address the problem of inaccurate citations in a scalable manner. An accurate NLP model could parse the citation-related text in a citing paper, automatically check whether that text is consistent with the cited reference paper, and flag the citation if an inconsistency is detected. Labeled data is needed for training and evaluating such a model. A significant barrier to development of NLP models for citation integrity is the lack of such labeled data. Studies of quotation errors in various medical domains are mostly limited to small datasets in specialized domains that are not publicly available (Davids *et al.* 2010, Awrey *et al.* 2011, Jergas and Baethge 2015, Pavlovic *et al.* 2021).

In this study, we aim to address this gap by developing an annotated corpus and NLP models toward automating citation integrity screening. Specifically, we make the following contributions: (i) we construct the first annotated corpus for assessing citation integrity targeting the biomedical literature; (ii) we report the first NLP models for citation integrity trained on the annotated corpus; and (iii) we make our corpus and models publicly available to enable further model development.

### 1.1 Related work

There is a long tradition of citation analysis studies, including qualitative, quantitative, and computational approaches (Smith 1981, Swales 1986, Bornmann and Daniel 2008, Zhang *et al.* 2013, Tahamtan and Bornmann 2019). While most work focuses on citation counts (Bornmann and Daniel 2008), there is increasing focus on describing and classifying relationships between the citing and reference papers (ie, *citation content analysis*) (Zhang *et al.* 2013, Tahamtan and Bornmann 2019), which aims to provide a more fine-grained, qualitative perspective on how an article is cited, including *citation function* (Spiegel-Rosing 1977, Teufel *et al.* 2009, Agarwal *et al.* 2010), *citation sentiment* (or polarity) (Athar 2014, Xu *et al.* 2015, Kilicoglu *et al.* 2019), and *citation influence* (Valenzuela *et al.* 2015, Zhu *et al.* 2015). We refer to reader to Iqbal *et al.* (2021) for a relatively recent survey of citation analysis research based on NLP and machine learning.

To our knowledge, citation integrity has not been studied from a NLP perspective, although NLP has been proposed as a potential tool for improving citation accuracy (Kilicoglu 2018). Citation integrity analysis differs from most citation analysis work in that it is a multi-document analysis task that requires consideration of both citing and reference articles. From this perspective, the most related NLP tasks are *citation-based scientific article summarization* (Qazvinian and Radev 2008, Jaidka *et al.* 2016, Cohan and Goharian 2017), and *claim verification* (Wadden *et al.* 2020). Citation-based scientific article summarization aims to link citation sentences (i.e. *citances*) with the relevant texts in a reference article to generate a summary of the reference article and has been studied within the context of CL-SciSumm shared task competitions (Jaidka *et al.* 2016, Chandrasekaran *et al.* 2019) which have focused on NLP literature. This task does not consider citation accuracy. Claim verification aims to identify “evidence from the research literature that supports or refutes a given scientific claim” (Wadden *et al.* 2020). Given a claim sentence, the goal of the task is to identify relevant evidence sentences (or *rationales*) from the abstracts of scientific publications and classify the evidence as supporting or refuting the claim or neutral to the claim (Support, Refute, Not Enough Information). Recently, several corpora have been developed focusing on health-related claims [SciFACT (Wadden *et al.* 2020), HealthVER (Sarrouti *et al.* 2021), and COVID-Fact (Kotonya and Toni 2020)]. This task is often formulated as a natural language inference task; first, relevant abstracts and rationale sentences are identified using information retrieval and a classification approach is used to make label predictions. Claim verification does not directly involve citations (or their integrity), although claims were generated by simplifying citation sentences in SciFACT (Wadden *et al.* 2020).

## 2 Materials and Methods

### 2.1 Data collection and processing

We collected 100 highly-cited research articles available in full text from the PubMed Central Open Access Subset (PMC-OA) to form our reference article set. To ensure a diverse set of articles, we searched PubMed for specific diseases (e.g. diabetes, COVID-19). Search strings and topic distribution of reference articles are provided in Supplementary Material. We leveraged OpCitance, a dataset of citation information collected from PubMed and PMC (Hsiao and Torvik 2023), to identify citation counts for the retrieved articles. One of the authors checked the articles for inclusion, ensuring that a variety of topics, article types (e.g. primary research study, review article), and study designs (e.g. meta-analysis, longitudinal study) were included.

The citing articles were sorted by the number of citations to the reference article, and a subset of articles citing the reference article multiple times were randomly selected for annotation. The number of citations was considered as a proxy for citation significance (Zhu *et al.* 2015).

After full texts of the citing articles were retrieved from the PMC-OA subset, we employed the process used in OpCitance to locate the citation marker in the citing article. For annotation, we extracted the paragraph containing the citation marker and highlighted the citation marker of interest in the paragraph.

### 2.2 Citation accuracy categories

**Figure 1.**
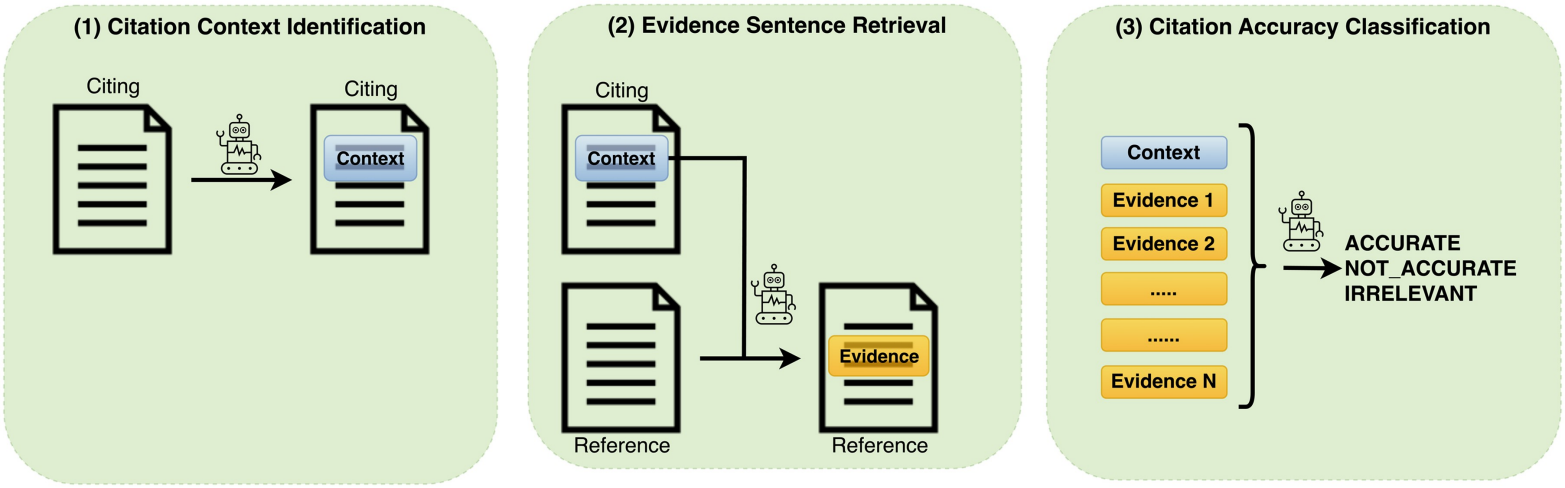
High-level illustration of the steps in the citation accuracy classification pipeline. Context rectangles represent citation context (in citing paper) and Evidence rectangles represent evidence segments (in reference paper).

Citation accuracy assessment is formulated as a three-part task (Fig. 1).

1. *Citation context identification:* In the citing paper, identify the text discussing aspects of the reference article.
2. *Evidence sentence retrieval:* In the reference paper, identify the texts (if any) that correspond to the citation context.
3. *Citation accuracy classification:* Based on the citation context and evidence sentences, determine whether the citation is supported by the evidence sentences (accurate) or is inconsistent with them (error). If there is an error, identify the error category (described below).

Our citation accuracy classification is based on and extends prior quotation error classifications (Luo *et al.* 2013, Jergas and Baethge 2015). These schemes distinguish major and minor errors, and provide further subcategories. In our classification, CONTRADICT, IRRELEVANT, and NOT_SUBSTANTIATE are major error categories and OVERSIMPLIFY, MISQUOTE, and INDIRECT minor error categories. In this work, we propose an additional minor error category, ETIQUETTE, and extend the definition of INDIRECT. We choose to annotate at this fine granularity as they are more descriptive and precise, compared to minor versus major error. We describe these categories below. We provide examples for each category in the Supplementary Material.

- ACCURATE: The citation context is consistent with an evidence segment in the reference article.
- CONTRADICT: The citation context contradicts a statement made in the reference article. This statement is annotated as the evidence segment.
- NOT_SUBSTANTIATE: The citation is relevant to the content of the reference article but the cited reference fails to substantiate all statements made in the citing paper.
- IRRELEVANT: There is no information in the reference article relevant to the citation.
- OVERSIMPLIFY: The findings of the reference article are oversimplified or overgeneralized.
- MISQUOTE: The numbers or percentages are misquoted.
- INDIRECT: The evidence segment includes a citation to other articles, indicating that the reference article is not the original source of the cited information. In prior work, this is generally applied when the reference articles are review articles; however, noting that this is a broader issue, we extended it to all types of reference articles.
- ETIQUETTE: This category, unique to our work, indicates that the citation style is ambiguous and it is unclear what is being cited from the reference article. This often occurs when the citation is part of a multi-citation (e.g. *[2–5]*), and may be a sign of “citation padding” (Fong and Wilhite 2017).

We note that the error categories above are listed in order of priority, i.e. contradictory errors are more problematic than etiquette errors. In cases when citations can be classified into multiple error categories (e.g. NOT_SUBSTANTIATE and INDIRECT), we select the higher priority error.

### 2.3 Corpus annotation

We used the brat annotation tool (Stenetorp *et al.* 2012) for annotation. For annotation, we provided annotators with the citation marker and the paragraph containing the citation marker from the citing article, and the full text of the reference article.

In the citing paragraph, the citation marker was pre-highlighted. As citation context, the annotators could annotate one or more sentences, as well as spans shorter than sentences. In the reference article, they were instructed to identify up to five evidence segments. Segments could be a single sentence, a section, or a paragraph, depending on the span relevant to the citation. For IRRELEVANT and ETIQUETTE labels, annotators indicated that there were no relevant or clear evidence segments.

Annotation was performed in three phases, and five annotators were involved in the annotation process. Annotators were graduate and undergraduate students in life sciences with experience in reading life sciences papers. Initial annotation guidelines were developed by the investigators, and they were extended and refined throughout the annotation process. The guidelines, including screenshots from brat interface, are provided in Supplementary Material.

In the first annotation phase, all 5 annotators annotated citations to the same 10 reference articles. Citations to one article were then reconciled by all annotators and the investigators. The remaining nine articles were split between three pairs (five annotators and one investigator), each pair reconciling citations to three articles. In the second phase, each annotator labeled citations to eight articles. They paired with each of the other four annotators for two articles, for a total of 20 articles. Each annotator pair then reconciled the citations that they both annotated. These steps were taken to ensure consistent understanding between annotators. In the third phase, each annotator individually annotated citations to 14 articles, for a total of 70 articles. After the third phase was completed, each annotator reviewed and corrected the annotations of another annotator, so each citation in this set was also double-checked. Finally, one of the annotators performed a final review to ensure consistent annotations.

### 2.4 Inter-annotator agreement calculation

We calculated pairwise inter-annotator agreement for each task: citation context identification, evidence segment identification, and citation accuracy classification. The first 30 reference articles (phases one and two) were included in calculation. Cohen’s kappa (κ) was used for all tasks, and the average pairwise agreement between annotators was calculated. For citation context and evidence segment identification, agreement was calculated at the sentence level. If an evidence segment annotation was at the paragraph or section level, all sentences in the paragraph/section are included as evidence sentences.

### 2.5 NLP models

#### 2.5.1 Citation context identification

We formulated the citation context identification problem as a sentence-level binary classification task. That is, given the citing paragraph and the citation marker, the goal of this task is to classify each sentence as part of the citation context or not. For this classification task, we fine-tuned the PubMedBERT model (Gu *et al.* 2021). Each sentence in the paragraph was paired with the citance to predict whether the sentence belonged in the citation context. The target citation marker is replaced with the special token [cit] (or [multi_cit] in the case of a citation marker that includes multiple citations) and other citations in the sentences are replaced with [other_cit]. The [cls] token representation is used for classification. The citance is automatically included in citation context. The experimental settings for this model are provided in Supplementary Material.

We compare this approach with a simple yet effective baseline, which simply uses the citance as the citation context.

#### 2.5.2 Evidence sentence retrieval

Identification of evidence sentences in the reference article is formulated as text pair classification (citation context and reference article pair). Because reference articles are longer than 512 tokens (the maximum number of tokens allowed by models like PubMedBERT Gu *et al.* 2021), for efficiency, we first identified relevant sentences from the reference article using a sentence retriever model and trained models that used the citation context along with the retrieved sentences to assess citation accuracy in a single step. We experimented with the following sentence retrieval approaches:

- *Title and abstract*: The baseline approach is to take the title and abstract of the reference article as evidence sentences. This is based on the intuition that the title and abstract discuss the most important contributions of the reference article, and these contributions are the most cited information. This is in line with scientific claim verification frameworks that rely on titles and abstracts as evidence (Wadden *et al.* 2020).
- *BM25 + MonoT5 reranker*: To identify the most semantically relevant sentences, we leverage the simple BM25 retrieval model (Robertson and Zaragoza 2009) to obtain the top 60 sentences and the MonoT5 reranker (Nogueira *et al.* 2020) to rerank these sentences. Finally, we use the top-*k* sentences as the evidence sentences. We experimented with different *k* values (5, 10, and 20).

In the cases using the BM25 + MonoT5 reranker, we experimented with three *query documents* as input for sentence retrieval: (i) citance only, (ii) the ground truth citation context sentences (i.e. all sentences annotated by annotators), and (iii) the ground truth citation context sentences along with the intervening sentences.

In addition to these methods that do not require specific training, we also used annotated evidence sentences for training a multi-task model that performs evidence sentence identification and citation accuracy classification simultaneously, which we describe in the next section.

#### 2.5.3 Citation accuracy classification using MultiVerS

We adopted a claim verification approach for citation accuracy classification. In claim verification, the input is a claim with a set of abstracts as possible evidence sources. We consider a single evidence source (reference article) rather than multiple abstracts at a time and full articles rather than abstracts. Furthermore, citation accuracy classes are different from claim verification classes (Support, Refute, Not Enough Information), although there is some overlap.

Due to these similarities, we leveraged a state-of-the-art claim verification model, MultiVerS (Wadden *et al.* 2022). For our baseline experiments, we used our training set to fine-tune the MultiVerS model trained on HealthVER (Sarrouti *et al.* 2021). We consolidated our labels into the three categories used by MultiVerS. Specifically, we mapped the ACCURATE and INDIRECT labels to Support; CONTRADICT, NOT_SUBSTANTIATE, OVERSIMPLIFY, MISQUOTE, and ETIQUETTE to Refute; and IRRELEVANT to Not Enough Information. INDIRECT, a minor error category, was merged with ACCURATE in training and evaluation, because the content of such citations often reflect the content of the reference articles accurately. The simplified categorization was adopted because in preliminary experiments we found that the models struggled with fine-grained error distinctions and because three-way classification still serves the overall goal of citation error identification. We name these classes ACCURATE, NOT_ACCURATE, and IRRELEVANT to better reflect the nature of the task.

MultiVerS incorporates both the claim and the evidence abstract and utilizes Longformer (Beltagy *et al.* 2020) as the encoder, which can process long sequences. The MultiVerS model performs two tasks in a multi-task setting: label prediction and rationale sentence selection. In our case, the citation context serves as the claim and evidence sentences identified in the previous step serve as the evidence abstract. We replaced citation markers in the citation context with special tokens as before. The representation of the entire citation context and evidence sentences were then fed into a three-way classification head for citation accuracy classification. We ignored the rationale sentence classifier by setting its loss function weight to 0, as preliminary experiments yielded poor performance. Rationale sentence classifier and the label classifier work in parallel and independently, so this does not impact the label classifier.

#### 2.5.4 In-context learning for citation accuracy classification

Generative large language models (LLMs) have been shown to be competitive for various NLP tasks when the task is specified by a natural language instruction (i.e. prompt) along with a few examples of the task (*in-context learning*) (Brown *et al.* 2020). Prompting the model to reason about the steps to arrive at a conclusion is shown to further improve performance for complex reasoning tasks (Wei *et al.* 2022). We evaluated two LLMs from OpenAI (GPT-3.5-turbo-0613 and GPT-4) for citation accuracy classification.

The models were provided with the citation context and evidence sentences identified in previous steps and prompted to return a prediction (ACCURATE, NOT_ACCURATE, or IRRELEVANT) along with their reasoning for the prediction. The prompt consists of a detailed task instruction along with descriptions of three classes, which is followed by four demonstrations selected from the training set (one each for ACCURATE and IRRELEVANT, and two for NOT_ACCURATE). The test case follows the demonstrations. XML-like tags and markdown elements are used to differentiate between the different parts of the prompt. The prompt template and an example are provided in the Supplementary Material. The models were evaluated in February, 2024.

### 2.6 Model evaluation

We report standard evaluation metrics, precision, recall, and their harmonic mean, F_1_ score, for citation context identification and accuracy classification tasks. For citation accuracy classification, we report both micro- and macro-averaged results. We assess whether the performance differences between the baseline MultiVerS model and the other models are statistically significant using McNemar’s test.

To evaluate whether sentence retrieval models are able to identify annotated evidence segments, we use recall@k and mean reciprocal rank (MRR). Recall@k is defined as the proportion of evidence sentences within the top k retrieved sentences. We use 1, 5, 10, and 20 as *k* values. MRR measures how far down the ranking the first relevant result is across multiple queries. We only consider the top 20 retrieved sentences for MRR. High recall@k and low MRR are preferred.

## 3 Results

### 3.1 Corpus statistics

A total of 3063 citation instances corresponding 3420 citation context sentences and 3791 evidence sentences were annotated (1.12 context and 1.24 evidence sentences per citation). The median number of citations per reference article was 27 (range: 11–74, IQR: 8.25). The median number of citing articles per reference article was 22 (range: 5–29, IQR: 6). Table 1 shows the label distribution in the dataset. The majority of citations were deemed accurate (60.82%). There were slightly more minor errors than major errors (21.16% versus 18.02%). Per paper, there was a statistically significant difference between the occurrence of minor and errors (Student’s *t*-test, *P* = .0085). On average, review articles had fewer citation errors than original articles (0.37 versus 0.4), although the difference was not statistically significant (Student’s *t*-test, *P* = .095). The average number of error types per paper was 0.52 in review articles and 0.54 in original papers (both ranging from 0 to 4).

**Table 1.** Distribution of citation accuracy labels.

Label	Total	Percentage
**ACCURATE**	**1863**	**60.82**
**MAJOR**	**552**	**18.02**
CONTRADICT	92	3.00
NOT_SUBSTANTIATE	243	7.93
IRRELEVANT	217	7.08
**MINOR**	**648**	**21.16**
MISQUOTE	38	1.24
OVERSIMPLIFY	111	3.62
INDIRECT	82	2.68
ETIQUETTE	417	13.61
**Total errors**	**1200**	39.18
**Total**	**3063**	100

Fine-grained error categories appear under their high-level groupings (MAJOR and MINOR errors), which are shown in bold.

### 3.2 Inter-annotator agreement

We calculated average pairwise inter-annotator agreement using Cohen’s *κ* for citation context, evidence sentence, and citation accuracy annotations over the first two phases of annotation (30 reference articles). We obtained high agreement on citation context annotation (*κ *= 0.96), as expected, as the agreement was measured at the sentence level and in most cases, citation context sentences are simply the citances. Agreement was lower on evidence sentence and accuracy label annotations, although the agreement improved in the second phase to fair agreement levels (evidence sentence: 0.20 in the first phase to 0.37 in the second; accuracy labels: 0.18–0.31).

### 3.3 NLP results

#### 3.3.1 Citation context identification

The citation context identification results are shown in Table 2. The baseline method (simply labeling the citance as the citation context) yields perfect precision, because citation context always includes the citance, and leads to our best results (0.94 F_1_). Fine-tuned PubMedBERT model was unable to improve on this simple baseline due to lower precision.

**Table 2.** Model performance for citation context classification.

Model	Precision	Recall	F_1_ score
Baseline (citance)	1.00	0.90	0.94
Fine-tuned BERT model	0.97	0.90	0.93

#### 3.3.2 Evidence sentence retrieval

Results for evidence sentence retrieval using the BM25 + MonoT5 reranker model are presented in Table 3. The results show that even with top-20 sentence retrieval, almost half of the sentences annotated as relevant were missed. Using different queries did not have a significant impact on the retrieval results, although using the full context performs slightly better.

**Table 3.** Recall@k and MRR for evidence sentence retrieval using BM25 + MonoT5 reranker.

Metric	Ground truth context	Citance	Full context
Recall@1	0.09	0.09	0.10
Recall@5	0.29	0.28	0.31
Recall@10	0.41	0.40	0.44
Recall@20	0.54	0.53	0.55
MRR	0.31	0.32	0.33

Columns correspond to different queries used for sentence retrieval.

#### 3.3.3 Citation accuracy classification

Based on the results for citation context classification and evidence sentence retrieval (Tables 2 and 3), we decided to use citance as citation context for citation accuracy classification.

The results for MultiVerS-based end-to-end models using various evidence sentence retrieval strategies as well as the results with GPT-based approach are presented in Table 4. Using title and abstract as the evidence is considered the baseline. In experiments using the top 20 sentences along with annotated evidence, annotated sentences are only used in training. GPT models use the top five sentences only, due to computational costs of longer evidence segments.

**Table 4.** Citation accuracy classification with citance as context and various evidence sentence retrieval strategies.

Evidence input	Label	F_1_	Micro/Macro-F_1_
*MultiVerS models*			
Title+abstract	ACC	0.69	
	N_ACC	0.38	0.56/0.43
	IRR	0.20	
Top 5 sentences	ACC	0.69	
	N_ACC	**0.43**	0.58/0.50
	IRR	0.37	
Top 10 sentences	ACC	0.67	
	N_ACC	0.41	0.56/0.48
	IRR	0.36	
Top 20 sentences	ACC	0.69	
	N_ACC	**0.43**	0.59/**0.52**
	IRR	0.42	
Top 20 sentences + annotated evidence	ACC	0.69	
	N_ACC	**0.43**	0.58/0.50
	IRR	0.38	
Oracle (gold citation + gold evidence)	ACC	0.80	
	N_ACC	0.57	0.75/0.78
	IRR	0.96	
Oracle (gold evidence)	ACC	0.79	
	N_ACC	0.52	0.73/0.75
	IRR	0.93	
*In-context learning*			
GPT-3.5-turbo	ACC	0.73	
	N_ACC	0.05	0.57/0.38
	IRR	0.34	
GPT-4	ACC	**0.80**	
	N_ACC	0.09	**0.65**/0.45
	IRR	**0.48**	

Top-*k* sentences are retrieved using BM25 + MonoT5 reranker. In-context learning uses top five sentences only. Best results are highlighted in bold. ACC: ACCURATE, N_ACC: NOT_ACCURATE, IRR: IRRELEVANT.

For MultiVerS models, the micro-averaged F_1_ score for all experiments ranged from 0.56 to 0.59 and the macro-averaged F_1_ from 0.43 to 0.52. The model that used as input the top 20 evidence sentences yielded the best overall results, while the baseline model yielded the lowest performance. However, the differences of model performances from the baseline were not statistically significant. The oracle model that used gold evidence sentences along with the citance yielded 0.73 micro-F_1_ and 0.75 macro-F_1_, while the model that also used gold citation contexts provided further improvement: 0.75 micro-F_1_ and 0.78 macro-F_1_. These model performances underscore the significance of precise identification of citation context, which can be shorter than a sentence, and evidence sentences.

In-context learning outperforms MultiVerS models in predicting ACCURATE citations. GPT-4 has a strong performance with 0.80 F_1_ score and 0.90 recall. However, these models perform poorly on the NOT_ACCURATE category, identifying only a few cases of this category leading to very low recall and F_1_. The performance differences between GPT-4 and the baseline MultiVerS and the best MultiVers model (top-20) were statistically significant (McNemar’s test: P≤.001 and P≤.05, respectively). The differences between GPT-3.5-turbo and the MultiverS models were not statistically significant.

Our primary focus is to identify citation errors (IRRELEVANT and NOT_ACCURATE). The best MultiVerS model (top-20) yielded the highest F_1_ for the NOT_ACCURATE class (0.43) and the second highest F_1_ for the IRRELEVANT class after GPT-4 (0.42 versus 0.48). Therefore, we consider this model our best model. We provide the full classification results (including precision and recall) and a more detailed error analysis for the best-performing model in Supplementary Material.

## 4 Discussion

### 4.1 Annotation

To our knowledge, our annotated corpus is the first focused on citation integrity in scientific literature. We created a sizable corpus using fine-grained error categories based on the literature, which enabled us to develop baseline NLP models.

Annotation of citation errors presented significant challenges, including lack of appropriate tools for annotating cross-document information, difficulty of locating evidence sentences in long full-text articles, and the often subtle nature of citation errors. We repurposed the brat annotation tool and post-processed the annotations to address the first problem. Annotation of precise evidence sentences is challenging because, in addition to the domain understanding required, multiple sentences can be relevant for a citation and annotators may annotate different relevant sentences, which leads to low inter-annotator agreement. To improve consistency, we provided detailed annotation guidelines and had annotators read the reference papers carefully before annotating the citations to them. While the consistency of annotators improved over time, it remained lower than desired. Different sentences in a reference article can relate to the citation in different ways. The annotators were instructed to prioritize major error types over minor types, but there was inconsistencies in following these instructions. All annotations were checked for consistency by a single annotator at the end of the process and some problematic cases were further reconciled by the senior investigator, which improved the overall data quality. It is also worth noting that other datasets which link citation sentences to reference article sentences often have low inter-annotator agreement (0.16–0.52) (Li *et al.* 2023). Citation error categories presented further challenges. For example, OVERSIMPLIFY and NOT_SUBSTANTIATE can be viewed as related categories differing in severity, but some subjectivity is involved in distinguishing them. This challenge led us to use a simplified three-way categorization in NLP development. However, in practice, fine-grained categories could be more useful compared to three-way categorization, as they can pinpoint the error more precisely. For example, IRRELEVANT category could suggest citation padding or, more seriously, research misconduct.

### 4.2 NLP models

Improving over the baseline of using the citance as citation context was difficult, partly because additional sentences annotated as citation context were rare. An additional experiment of 5-shot in-context learning with GPT-3.5-turbo also did not improve results over baseline (0.89 F_1_ score). Using gold citation contexts in addition to gold evidence segments did improve citation accuracy classification (Table 4), suggesting that span-level citation context classification could benefit the accuracy classification task.

We chose the MultiVerS model for our task, due to its state-of-the-art performance for claim verification and the similarity between this task and ours. Initially, we used their multi-task formulation to both identify the evidence sentences and label accuracy. Because we considered much longer texts and evidence sentence annotations could not be guaranteed to be exhaustive, however, this approach yielded poor performance. A simplified formulation that used BM25 + MonoT5 reranker for sentence retrieval and MultiVerS for label classification only significantly improved results. This is also more efficient, as no sentence retrieval model needs to be trained. Our results with MultiVerS are overall lower than the reported performance on claim verification datasets such as SciFACT, which underscores the challenging nature of citation accuracy classification.

Among two generative LLMs we experimented with, GPT-4 performed significantly better than GPT-3.5-turbo. GPT-4 performed even better than the fine-tuned MultiVerS models in recognizing ACCURATE and IRRELEVANT citations. On the other hand, its performance on NOT_ACCURATE class is quite poor, which makes it unsuitable for practical use as this is arguably the most important label. However, we only experimented with slight variations on a manual prompt and better prompting strategies, specifically focusing on NOT_ACCURATE, could yield better results. We performed additional experiments (fine-tuning, binary task formulation) with GPT-3.5-turbo, which showed mixed results (the results in Supplementary Material).

The results raise the question of whether it would be worth training models for identifying evidence sentences. Retrieving top-20 sentences with BM25 + MonoT5 reranker has a recall of 0.53, which indicates significant differences between the annotated and automatically retrieved sentences. Using gold evidence sentences (oracle in Table 4) yields 14% and 23% points higher micro- and macro-F_1_, respectively. Examining the model predictions, we find that in cases where some gold evidence sentences are retrieved by the sentence retriever, 63% of the predictions are correct. On the other hand, when no gold evidence sentence is retrieved, 51% are correctly predicted, which suggests that a trained sentence retriever could further improve the results.

### 4.3 Limitations

Our annotation yielded fair inter-annotator agreement for evidence sentence annotation and citation accuracy classification. Hiring annotators with specific expertise in the topic areas of the reference papers could have improved agreement, but was not feasible. To improve data quality, we took additional steps, such as multiple rounds of verification for consistency. We did not consider whether or not a citation is significant for the citing article; it may be less important to assess the citation accuracy if a given citation is perfunctory. We excluded cases in which the relevant evidence was in tables/figures or in Supplementary Material, which is fairly common.

The performance of our models on citation errors was low, although this is not entirely surprising given the challenging nature of the task even for humans. We focused on a three-way classification, whereas our annotation consists of fine-grained labels. Based on our preliminary experiments, we decided to use a pre-trained model for sentence retrieval, but further improvements might be possible by training a dense retriever using the annotated evidence sentences.

## 5 Conclusions

We presented the first publicly available corpus that focuses on citation quotation errors and reported NLP models for this task. The corpus could facilitate further model development and also serves as a challenging NLP corpus. While the performance of the models are not yet high for practical use, with further improvements, such models could help authors in improving their citation practices and journals in scrutinizing submitted manuscripts more effectively for citation integrity errors. In the long term, we envision NLP-based tools that can pinpoint and help raise awareness around poor citation practices, contributing to increased rigor in scholarly communication. Because underlying NLP models are unlikely to be perfect, such tools should be considered as supplementary aids to human judgment.

In future work, we aim to extend our models to recognize fine-grained error labels, which are more informative, in addition to three-way classification. We plan to explore data augmentation for less frequent error categories. Future work also needs to consider the cases where the citations refer to tables/figures, or Supplementary Materials, although processing non-standard supplementary files for accuracy classification is likely to be challenging.

## Supplementary Material

btae420_Supplementary_Data
