# Supplementary material for: Assessing citation integrity in biomedical publications: corpus annotation and NLP models
Source: Bioinformatics. 2024 Jun 26;40(7):btae420. doi: 10.1093/bioinformatics/btae420 (PMC11231046; doi:10.1093/bioinformatics/btae420)
Supplement: btae420_Supplementary_Data [file btae420_supplementary_data.docx]

Supplementary Material for *Assessing Citation Integrity in Biomedical Publications: Corpus Annotation and NLP Models*

M. Janina Sarol, Shufan Ming, Shruthan Radhakrishna, Jodi Schneider, and Halil Kilicoglu

Table of Contents

1. [Topical distribution of reference articles](#A)
2. [Search strings](#B)
3. [Citation accuracy categories](#C)
4. [Annotation guidelines](#D)
5. [Experimental settings](#E)
6. [Prompt structure](#F)
7. [Full citation accuracy classification results](#G)
8. [Error analysis](#H)
9. [GPT-3.5-turbo experiments](#I)
10. Topical distribution of reference articles

Table S1. Topical distribution of reference articles included in the dataset.

| **Main Topic** | **Number of Reference Articles** |
| --- | --- |
| COVID-19 | 31 |
| Diabetes/obesity | 19 |
| Cancer | 12 |
| AIDS | 11 |
| Alzheimer’s disease | 5 |
| Autism | 5 |
| Myocardial infarction/stroke | 5 |
| Parkinson’s disease | 5 |
| Depression | 2 |
| Inflammatory bowel disease | 3 |
| Rheumatoid arthritis | 1 |
| Multiple diseases | 1 |
| Total | 100 |

1. Search strings

Searches were conducted on July 31, 2022. Note that results related to some health conditions were ultimately not used (e.g., asthma).

- **Alzheimer’s disease** (3138 abstracts retrieved)

**Search**: pubmed pmc open access[filter] AND Research Support, U.S. Gov’t, P.H.S.[Publication Type] AND alzheimer

**Filters**: 2010 - 2019

("pubmed pmc open access"[Filter] AND "research support, u s gov t, p h s"[Publication Type] AND ("alzheime s"[All Fields] OR "alzheimer disease"[MeSH Terms] OR ("alzheimer"[All Fields] AND "disease"[All Fields]) OR "alzheimer disease"[All Fields] OR "alzheimer"[All Fields] OR "alzheimers"[All Fields] OR "alzheimer s"[All Fields] OR "alzheimers s"[All Fields])) AND (2010:2019[pdat])

- **COVID-19** (7401 abstracts)

**Search**: pubmed pmc open access[filter] AND Research Support, U.S. Gov’t, P.H.S.[Publication Type] AND COVID-19

**Filters**: from 2019 - 2022

("pubmed pmc open access"[Filter] AND "research support, u s gov t, p h s"[Publication Type] AND ("covid 19"[All Fields] OR "covid 19"[MeSH Terms] OR "covid 19 vaccines"[All Fields] OR "covid 19 vaccines"[MeSH Terms] OR "covid 19 serotherapy"[All Fields] OR "covid 19 serotherapy"[Supplementary Concept] OR "covid 19 nucleic acid testing"[All Fields] OR "covid 19 nucleic acid testing"[MeSH Terms] OR "covid 19 serological testing"[All Fields] OR "covid 19 serological testing"[MeSH Terms] OR "covid 19 testing"[All Fields] OR "covid 19 testing"[MeSH Terms] OR "sars cov 2"[All Fields] OR "sars cov 2"[MeSH Terms] OR "severe acute respiratory syndrome coronavirus 2"[All Fields] OR "ncov"[All Fields] OR "2019 ncov"[All Fields] OR (("coronavirus"[MeSH Terms] OR "coronavirus"[All Fields] OR "cov"[All Fields]) AND 2019/11/01:3000/12/31[Date - Publication]))) AND (2019:2022[pdat])

- **HIV or AIDS** (8548 abstracts)

**Search**: pubmed pmc open access[filter] AND Research Support, U.S. Gov’t, P.H.S.[Publication Type] AND (HIV or AIDS)

**Filters**: from 2010 - 2019

("pubmed pmc open access"[Filter] AND "research support, u s gov t, p h s"[Publication Type] AND ("hiv"[MeSH Terms] OR "hiv"[All Fields] OR ("acquired immunodeficiency syndrome"[MeSH Terms] OR ("acquired"[All Fields] AND "immunodeficiency"[All Fields] AND "syndrome"[All Fields]) OR "acquired immunodeficiency syndrome"[All Fields] OR "aids"[All Fields]))) AND (2010:2019[pdat])

- **Asthma** (1112 abstracts)

**Search**: pubmed pmc open access[filter] AND Research Support, U.S. Gov’t, P.H.S.[Publication Type] AND asthma

**Filters**: 2010 - 2019

("pubmed pmc open access"[Filter] AND "research support, u s gov t, p h s"[Publication Type] AND ("asthma"[MeSH Terms] OR "asthma"[All Fields] OR "asthmas"[All Fields] OR "asthma s"[All Fields])) AND (2010:2019[pdat])

- **Autism** (1268 abstracts)

**Search**: pubmed pmc open access[filter] AND Research Support, U.S. Gov’t, P.H.S.[Publication Type] AND autism

**Filters**: from 2010 - 2019

("pubmed pmc open access"[Filter] AND "research support, u s gov t, p h s"[Publication Type] AND ("autism s"[All Fields] OR "autisms"[All Fields] OR "autistic disorder"[MeSH Terms] OR ("autistic"[All Fields] AND "disorder"[All Fields]) OR "autistic disorder"[All Fields] OR "autism"[All Fields])) AND (2010:2019[pdat])

- **Stroke** (2623 abstracts)

**Search**: pubmed pmc open access[filter] AND Research Support, U.S. Gov’t, P.H.S.[Publication Type] AND stroke

**Filters**: from 2010 - 2019

("pubmed pmc open access"[Filter] AND "research support, u s gov t, p h s"[Publication Type] AND ("stroke"[MeSH Terms] OR "stroke"[All Fields] OR "strokes"[All Fields] OR "stroke s"[All Fields])) AND (2010:2019[pdat])

- **Diabetes** (9303 abstracts)

**Search**: pubmed pmc open access[filter] AND Research Support, U.S. Gov’t, P.H.S.[Publication Type] AND diabetes

**Filters**: from 2010 - 2019

("pubmed pmc open access"[Filter] AND "research support, u s gov t, p h s"[Publication Type] AND ("diabete"[All Fields] OR "diabetes mellitus"[MeSH Terms] OR ("diabetes"[All Fields] AND "mellitus"[All Fields]) OR "diabetes mellitus"[All Fields] OR "diabetes"[All Fields] OR "diabetes insipidus"[MeSH Terms] OR ("diabetes"[All Fields] AND "insipidus"[All Fields]) OR "diabetes insipidus"[All Fields] OR "diabetic"[All Fields] OR "diabetics"[All Fields] OR "diabets"[All Fields])) AND (2010:2019[pdat])

- **Acid reflux** (68 abstracts)

**Search**: pubmed pmc open access[filter] AND Research Support, U.S. Gov’t, P.H.S.[Publication Type] AND acid reflux

**Filters**: from 2010 - 2019

("pubmed pmc open access"[Filter] AND "research support, u s gov t, p h s"[Publication Type] AND ("gastroesophageal reflux"[MeSH Terms] OR ("gastroesophageal"[All Fields] AND "reflux"[All Fields]) OR "gastroesophageal reflux"[All Fields] OR ("acid"[All Fields] AND "reflux"[All Fields]) OR "acid reflux"[All Fields])) AND (2010:2019[pdat])

- **Heart failure** (1372 abstracts)

**Search**: pubmed pmc open access[filter] AND Research Support, U.S. Gov’t, P.H.S.[Publication Type] AND heart failure

**Filters**: from 2010 - 2019

("pubmed pmc open access"[Filter] AND "research support, u s gov t, p h s"[Publication Type] AND ("heart failure"[MeSH Terms] OR ("heart"[All Fields] AND "failure"[All Fields]) OR "heart failure"[All Fields])) AND (2010:2019[pdat])

- **Hypercholesterolemia** (171 abstracts)

**Search**: pubmed pmc open access[filter] AND Research Support, U.S. Gov’t, P.H.S.[Publication Type] AND hypercholesterolemia

**Filters**: from 2010 - 2019

("pubmed pmc open access"[Filter] AND "research support, u s gov t, p h s"[Publication Type] AND ("hypercholesterolaemia"[All Fields] OR "hypercholesterolemia"[MeSH Terms] OR "hypercholesterolemia"[All Fields] OR "hypercholesterolaemias"[All Fields] OR "hypercholesterolemias"[All Fields])) AND (2010:2019[pdat])

- **Hypertension** (2699 abstracts)

**Search**: pubmed pmc open access[filter] AND Research Support, U.S. Gov’t, P.H.S.[Publication Type] AND hypertension

**Filters**: from 2010 - 2019

("pubmed pmc open access"[Filter] AND "research support, u s gov t, p h s"[Publication Type] AND ("hypertense"[All Fields] OR "hypertension"[MeSH Terms] OR "hypertension"[All Fields] OR "hypertension s"[All Fields] OR "hypertensions"[All Fields] OR "hypertensive"[All Fields] OR "hypertensive s"[All Fields] OR "hypertensives"[All Fields])) AND (2010:2019[pdat])

- **Inflammatory bowel disease** (688 abstracts)

**Search**: pubmed pmc open access[filter] AND Research Support, U.S. Gov’t, P.H.S.[Publication Type] AND inflammatory bowel disease

**Filters**: from 2010 - 2019

("pubmed pmc open access"[Filter] AND "research support, u s gov t, p h s"[Publication Type] AND ("inflammatory bowel diseases"[MeSH Terms] OR ("inflammatory"[All Fields] AND "bowel"[All Fields] AND "diseases"[All Fields]) OR "inflammatory bowel diseases"[All Fields] OR ("inflammatory"[All Fields] AND "bowel"[All Fields] AND "disease"[All Fields]) OR "inflammatory bowel disease"[All Fields])) AND (2010:2019[pdat])

- **Depression** (2760 abstracts)

**Search**: pubmed pmc open access[filter] AND Research Support, U.S. Gov’t, P.H.S.[Publication Type] AND depression

**Filters**: from 2010 - 2019

("pubmed pmc open access"[Filter] AND "research support, u s gov t, p h s"[Publication Type] AND ("depressed"[All Fields] OR "depression"[MeSH Terms] OR "depression"[All Fields] OR "depressions"[All Fields] OR "depression s"[All Fields] OR "depressive disorder"[MeSH Terms] OR ("depressive"[All Fields] AND "disorder"[All Fields]) OR "depressive disorder"[All Fields] OR "depressivity"[All Fields] OR "depressive"[All Fields] OR "depressively"[All Fields] OR "depressiveness"[All Fields] OR "depressives"[All Fields])) AND (2010:2019[pdat])

- **Breast cancer** (5346 articles)

**Search**: pubmed pmc open access[filter] AND Research Support, U.S. Gov’t, P.H.S.[Publication Type] AND breast cancer

**Filters**: from 2010 - 2019

("pubmed pmc open access"[Filter] AND "research support, u s gov t, p h s"[Publication Type] AND ("breast neoplasms"[MeSH Terms] OR ("breast"[All Fields] AND "neoplasms"[All Fields]) OR "breast neoplasms"[All Fields] OR ("breast"[All Fields] AND "cancer"[All Fields]) OR "breast cancer"[All Fields])) AND (2010:2019[pdat])

- **Colon cancer** (1283 abstracts)

**Search**: pubmed pmc open access[filter] AND Research Support, U.S. Gov’t, P.H.S.[Publication Type] AND colon cancer

**Filters**: from 2010 - 2019

("pubmed pmc open access"[Filter] AND "research support, u s gov t, p h s"[Publication Type] AND ("colonic neoplasms"[MeSH Terms] OR ("colonic"[All Fields] AND "neoplasms"[All Fields]) OR "colonic neoplasms"[All Fields] OR ("colon"[All Fields] AND "cancer"[All Fields]) OR "colon cancer"[All Fields])) AND (2010:2019[pdat])

- **Migraine** (140 abstracts)

**Search**: pubmed pmc open access[filter] AND Research Support, U.S. Gov’t, P.H.S.[Publication Type] AND migraine

**Filters**: from 2010 - 2020

("pubmed pmc open access"[Filter] AND "research support, u s gov t, p h s"[Publication Type] AND ("migrain"[All Fields] OR "migraine disorders"[MeSH Terms] OR ("migraine"[All Fields] AND "disorders"[All Fields]) OR "migraine disorders"[All Fields] OR "migraine"[All Fields] OR "migraines"[All Fields] OR "migraine s"[All Fields] OR "migraineous"[All Fields] OR "migrainers"[All Fields] OR "migrainous"[All Fields])) AND (2010:2020[pdat])

- **Myocardial infarction** (1023 abstracts)

**Search**: pubmed pmc open access[filter] AND Research Support, U.S. Gov’t, P.H.S.[Publication Type] AND myocardial infarction

**Filters**: from 2010 - 2019

("pubmed pmc open access"[Filter] AND "research support, u s gov t, p h s"[Publication Type] AND ("myocardial infarction"[MeSH Terms] OR ("myocardial"[All Fields] AND "infarction"[All Fields]) OR "myocardial infarction"[All Fields])) AND (2010:2019[pdat])

- **Obesity** (5697 abstracts)

**Search**: pubmed pmc open access[filter] AND Research Support, U.S. Gov’t, P.H.S.[Publication Type] AND obesity

**Filters**: from 2010 - 2019

("pubmed pmc open access"[Filter] AND "research support, u s gov t, p h s"[Publication Type] AND ("obeses"[All Fields] OR "obesity"[MeSH Terms] OR "obesity"[All Fields] OR "obese"[All Fields] OR "obesities"[All Fields] OR "obesity s"[All Fields])) AND (2010:2019[pdat])

- **Parkinson’s disease** (1413 abstracts)

**Search**: pubmed pmc open access[filter] AND Research Support, U.S. Gov’t, P.H.S.[Publication Type] AND parkinson

**Filters**: from 2010 - 2019

("pubmed pmc open access"[Filter] AND "research support, u s gov t, p h s"[Publication Type] AND ("parkinson disease"[MeSH Terms] OR ("parkinson"[All Fields] AND "disease"[All Fields]) OR "parkinson disease"[All Fields] OR "parkinsons"[All Fields] OR "parkinson"[All Fields] OR "parkinson s"[All Fields] OR "parkinsonian disorders"[MeSH Terms] OR ("parkinsonian"[All Fields] AND "disorders"[All Fields]) OR "parkinsonian disorders"[All Fields] OR "parkinsonism"[All Fields] OR "parkinsonisms"[All Fields] OR "parkinsons s"[All Fields])) AND (2010:2019[pdat])

- **Prostate cancer** (2217 abstracts)

**Search**: pubmed pmc open access[filter] AND Research Support, U.S. Gov’t, P.H.S.[Publication Type] AND prostate cancer

**Filters**: from 2010 - 2019

("pubmed pmc open access"[Filter] AND "research support, u s gov t, p h s"[Publication Type] AND ("prostatic neoplasms"[MeSH Terms] OR ("prostatic"[All Fields] AND "neoplasms"[All Fields]) OR "prostatic neoplasms"[All Fields] OR ("prostate"[All Fields] AND "cancer"[All Fields]) OR "prostate cancer"[All Fields])) AND (2010:2019[pdat])

- **Rheumatoid arthritis** (589 abstracts)

**Search**: pubmed pmc open access[filter] AND Research Support, U.S. Gov’t, P.H.S.[Publication Type] AND rheumatoid arthritis

**Filters**: from 2010 - 2019

("pubmed pmc open access"[Filter] AND "research support, u s gov t, p h s"[Publication Type] AND ("arthritis, rheumatoid"[MeSH Terms] OR ("arthritis"[All Fields] AND "rheumatoid"[All Fields]) OR "rheumatoid arthritis"[All Fields] OR ("rheumatoid"[All Fields] AND "arthritis"[All Fields]))) AND (2010:2019[pdat])

- **Sleeplessness** (54 abstracts)

**Search**: pubmed pmc open access[filter] AND Research Support, U.S. Gov’t, P.H.S.[Publication Type] AND sleeplessness

**Filters**: from 2010 - 2019

("pubmed pmc open access"[Filter] AND "research support, u s gov t, p h s"[Publication Type] AND ("sleep initiation and maintenance disorders"[MeSH Terms] OR ("sleep"[All Fields] AND "initiation"[All Fields] AND "maintenance"[All Fields] AND "disorders"[All Fields]) OR "sleep initiation and maintenance disorders"[All Fields] OR "sleeplessness"[All Fields] OR "sleepless"[All Fields])) AND (2010:2019[pdat])

- **Hepatitis B** (317 abstracts)

**Search**: pubmed pmc open access[filter] AND Research Support, U.S. Gov’t, P.H.S.[Publication Type] AND hepatitis b

**Filters**: from 2010 - 2019

("pubmed pmc open access"[Filter] AND "research support, u s gov t, p h s"[Publication Type] AND ("hepatitis b"[MeSH Terms] OR "hepatitis b"[All Fields])) AND (2010:2019[pdat])

# Citation Accuracy Categories

PMC identifiers of articles are in parentheses, citation markers of interest are underlined, and for error cases, explanations are provided.

- **ACCURATE**: The citation context is consistent with an evidence segment in the reference article.

***Citation context***: Patients receiving remdesivir had a shorter time to recovery (defined as meeting the criteria for the top 3 categories on an 8-point ordinal scale) compared with those receiving placebo (median time to recovery, 10 [95% CI, 9–11] vs 15 [95% CI, 13–18] days; rate ratio for recovery, 1.29 [95% CI, 1.12–1.49]; P $<$ .001) [11]. (PMC8244650)

***Evidence segment***: Those who received remdesivir had a median recovery time of 10 days (95% confidence interval [CI], 9 to 11), as compared with 15 days (95% CI, 13 to 18) among those who received placebo (rate ratio for recovery, 1.29; 95% CI, 1.12 to 1.49; P $<$ 0.001, by a log-rank test). (PMC7262788)

- **CONTRADICT**: The citation context contradicts a statement made in the reference article. This statement is annotated as the evidence segment.

***Citation context***: This is coherent with the fact that hACE2 expression were not observed in the gut of the mice used in that study [37]. (PMC9573230)

***Evidence segment***: In the gastrointestinal tract of K18-hACE2 mice, hACE2 was expressed most abundantly in the colon, which correlated with infection seen at later time points. (PMC7578095)

***Explanation***: The colon is part of the gut.

- **NOT_SUBSTANTIATE**: The citation is relevant to the content of the reference article but the cited reference fails to substantiate all statements made in the citing paper.

***Citation context:*** Most studies have focused on the prevalence of high BMI, associating it with increased risk of conditions such as coronary heart disease, stroke, diabetes, and cancer.^6^ (PMC4753952)

***Evidence segment:*** Metabolic mediators of the effects of body-mass index, overweight, and obesity on coronary heart disease and stroke: a pooled analysis of 97 prospective cohorts with 1·8 million participants (PMC3959199)

***Explanation*:** BMI-cancer association is not examined in the reference paper.

- **IRRELEVANT**: There is no information in the reference article relevant to the citation.

***Citation context***: All analyses were performed using R software version 3.0.2 (library survival) and Stata 13.0 (College Station, TX) [29]. (PMC3867319)

***Reference article title***: Closing the Gap: Increases in Life Expectancy among Treated HIV-Positive Individuals in the United States and Canada (PMC4369842)

***Explanation:*** R or Stata is not mentioned in the reference article. This is likely an instance of authors citing the incorrect reference. The previous reference in the bibliography (28) is to the R software.

- ***OVERSIMPLIFY:*** The findings of the reference article are oversimplified or overgeneralized.

***Citation context:*** The introduction of combination antiretroviral therapy (cART) has changed HIV-1 infection from a life-threatening disease to a manageable chronic condition, and life expectancy of people living with HIV (PLWH) is approaching that of non-infected individuals [1]. (PMC6375138)

***Evidence segment:*** A 20-year-old HIV-positive adult on ART in the U.S. or Canada is expected to live into their early 70s, a life expectancy approaching that of the general population. (PMC3867319)

***Explanation:*** Age and region qualifications are discarded in the citing article.

- ***MISQUOTE:*** The numbers or percentages are misquoted.

***Citation context:*** Recent reviews show 32% loss to follow-up during this pre-treatment period despite eligibility for ART [12] (PMC3210087)

***Evidence segment:*** The rate of early mortality and loss to follow-up, which itself portends mortality for many, averages 23% across the region [2]. (PMC3139665)

***Explanation:*** 23% is misquoted as 32%.

- ***INDIRECT:*** The evidence segment includes a citation to other articles, indicating that the reference article is not the original source of the information cited. In prior characterizations, this is generally applied when the reference articles are review articles; however, noting that this is a broader issue, we extended it to all types of reference articles.

***Citation context:*** It is known that glycosylation has crucial roles in viral pathobiology, such as protein proper folding and viral tropism [20]. (PMC7199903)

***Evidence segment***: Viral glycosylation has wide-ranging roles in viral pathobiology, including mediating protein folding and stability and shaping viral tropism (9). (PMC8196891)

***Explanation:*** The information about glycosylation roles are not originally from the reference article.

- ***ETIQUETTE***: This category, unique to our work, indicates that the citation style is ambiguous and it is unclear what is being cited from the reference article. This often occurs when the citation is part of multi-citation, and may be a sign of “citation padding”.

***Citation context***: The iSPNs are enriched in enkephalin and D2 type dopamine receptors, project exclusively to the GPe, and play a role in suppression of unwanted movements (Albin et al., 1989; Sano et al., 2003; Kravitz et al., 2010; Cui et al., 2013; Freeze et al., 2013). (PMC4396197)

***Explanation:*** The underlined reference article only discusses the role of iSPNs in suppressing unwanted movements. It is unclear whether the rest of the cited articles substantiate the other information in the citation context.

We note that the error categories are listed in order of priority, i.e., CONTRADICT errors are more problematic than ETIQUETTE errors. In cases when citations can be classified into multiple error categories (e.g., NOT_SUBSTANTIATE and INDIRECT), we select the higher priority error. An example of a multi-error citation context is shown below:

***Citation context:*** Diabetic Macular Oedema (DMO) is one of the leading causes of blindness in the working-age population [1, 2].

***Evidence segment:*** Diabetic retinopathy (DR) is the leading cause of blindness among working-aged adults around the world (1). (PMC3322721)

***Explanation:*** In the example above, the citation contains two errors: first, the information is unsubstantiated (NOT_SUBSTANTIATE) since the evidence segment discusses diabetic retinopathy and not diabetic macular oedema. Second, the relevant evidence segment itself contains a citation, indicating that the information originally comes from another article (INDIRECT).

#

# Annotation Guidelines

In this project, we will annotate citation behavior in biomedical publications. In particular, we are interested in whether or not, when authors cite a paper in their manuscript, they are doing this in a way that is consistent with the findings of the cited paper (i.e., whether their citation is accurate). These annotations will be used to build machine learning models that can support humans in making such judgements.

**Definitions:**

| ***Citing paper***: A paper that cites another paper. |
| --- |
| ***Reference paper:*** A paper that is cited by another paper (also cited paper). |
| ***Citation marker:*** A string that represents the citation to the reference paper ([1], (2-5), Smith et al. (2015), etc.). A citation marker can contain a single citation or a multi-citation such as (2-5) above which includes citations to articles 2, 3, 4, and 5. |
| ***Citation sentence:*** Sentence with the citation marker. |
| ***Citation context:*** The sentence(s) or clauses in a citing paper that contain the citation and discuss some aspect of the reference paper. These are spans that help us understand the contribution of a particular reference paper to the citing paper. The citation context almost always includes the citation sentence (or some clause in it). It may also include some sentences surrounding the citation sentence. |
| ***Evidence segment:*** The portions of the reference paper that the authors of the citing paper discuss when they cite the reference paper. When citing a reference paper, authors of the citing paper can quote it verbatim, refer to a sentence or passage in it, or give a general reference to the entire paper. Our goal is to link citation contexts in the citing paper with up to five evidence segments in the reference paper to support citation accuracy judgements. |
| ***Citation accuracy:*** If the content of the citation context is consistent with the corresponding evidence segments, the citation is accurate. If the citation could mislead the reader but is not sufficiently serious to fundamentally alter the meaning of the source, it has a minor error. If the citation seriously misrepresents the original source or no related evidence segment is found, it has a major error. |

**Annotation task:**

You will be provided a citing-reference paper pair. There are three tasks in annotation:

1. In the citing paper, identify and annotate the text that discusses some aspect of the reference paper (i.e., citation context).
2. In the reference paper, identify and annotate the texts that best correspond to the citation context identified in step 1 (i.e., evidence segments).
3. Based on the citation context and evidence segment annotations (steps 1 and 2), annotate whether the citation is supported by the evidence segments (accurate) or is inconsistent with them (error). If there is an error, identify the error category (described below).


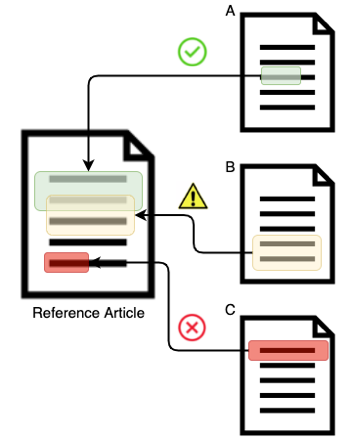
As reference papers, we use highly cited biomedical publications. For each reference paper, you will be provided at least 20 articles that cite it. More specifically, you will be provided a paragraph in the citing paper where the reference article is cited and the citation marker to the reference paper is highlighted for annotation convenience.

The annotation will be carried out in three steps: a) citation context annotation, and b) evidence segment annotation, and citation accuracy assessment. These tasks are discussed below.

Before the annotation starts, we will share the reference articles and ask you to read them in full and write a short summary to assess your understanding. We expect that this step will be helpful to understand what to look for in citation context analysis and reduce the need to repeatedly consult the reference articles.

Figure 1. An illustration of the annotation process. A, B, C are citing articles. Citation in A is accurate. Citation in B has a minor citation error, while one from C has a major error. The citation contexts in A, B, C and the evidence segments in the reference article are highlighted.

**Annotation tool:**

We use the *brat* tool^[[1]](#footnote-1)^ for annotation. The annotation URL is: <URL>. Brat works best on Chrome. To annotate, a username/password is needed (*brat/annotate*). To login, hover over the document bar at the top of the page, which will display the login button on the right, as shown below. You can view annotations without logging in.


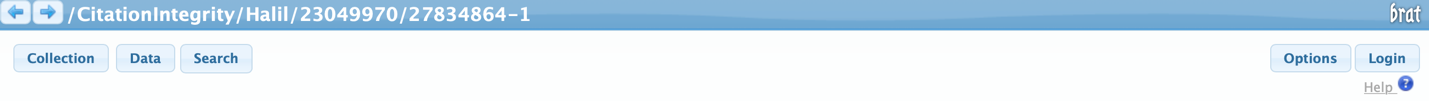


Each annotator has their own annotation folder and should only annotate in that folder. Under the folder with your name, you will find sub-folders which have the reference article IDs as their names. Under each sub-folder, you will find annotation files for that reference article. Each annotation file includes a single citation marker from a citing article to the reference article. Each sub-folder will have the following structure:

ReferenceArticleID

- CitingArticleID1_1
- CitingArticleID1_2
- CitingArticleID2_1
- CitingArticleID2_2
- CitingArticleID3_1, ….

The structure above indicates that there are two citations to the reference article in CitingArticleID1 and CitingArticleID2 and one citation in CitingArticleID3. In addition to the paragraph from the citing article that has the citation of interest, each file (e.g., CitingArticleID1-1) will also contain reference article delimited by the line ****. An example screenshot is below. In this example, the reference article is PMC23049970 and the citation under consideration is the first one from the article PMC27834964 (hence the *path /CitationIntegrity/Halil/ PMC23049970/PMC27834864_1*)^^[[2]](#footnote-2)^^. The citation marker “[42]” is highlighted. The top part of the file is the paragraph from the citing article with the ID PMC27834964 and the bottom part shows the title and the abstract of the reference article PMC23049970.


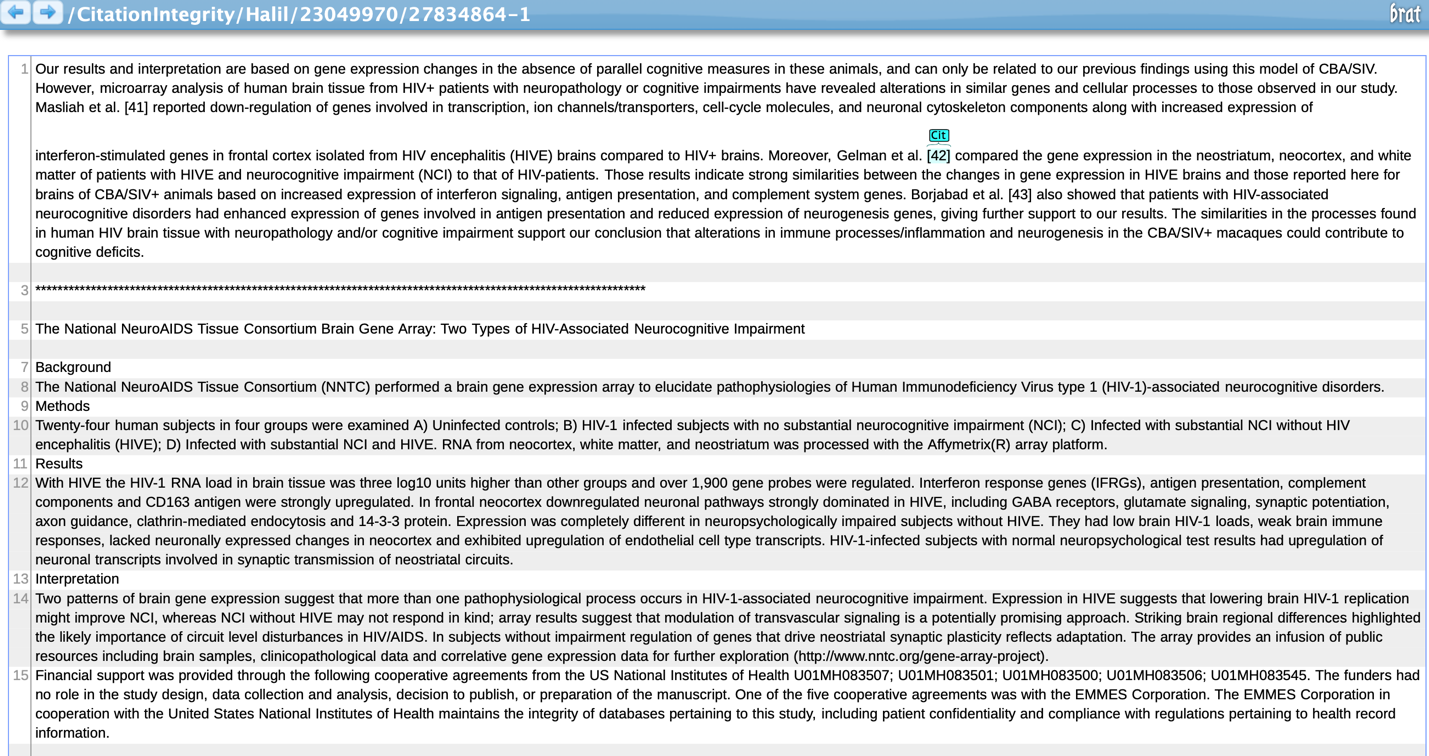


**Step 1. Citation context annotation:**

In this step, you will annotate the context around the citation marker that discusses the reference article. As mentioned above, the citation marker is already highlighted (labeled as *Citation/MultiCitation* or *Cit/MultiCit* in abbreviated form, respectively). The corresponding citation context can be:

- the sentence containing the citation marker
- a fragment of the sentence containing the citation marker
- a set of sentences (consecutive or non-consecutive) surrounding the citation marker

This step consists of two sub-steps:

- Select and annotate a sentence or fragment in a sentence as a Context object.
- Repeat the previous steps if there are multiple sentences that are relevant as citation context. *NOTE: Never annotate more than one sentence in a single Context object. Instead use multiple Context objects.*

**Context annotation examples:**

In the citing article above (PMC27834864), the reference article (PMC23049970) is cited twice. Two paragraphs where these citations appear are shown below (note that these will be in two different files in annotation, each file contains a single citation instance). Citation markers are highlighted in blue and the citation contexts in green.

Citation 1:

*Our results and interpretation are based on gene expression changes in the absence of parallel cognitive measures in these animals, and can only be related to our previous findings using this model of CBA/SIV. However, microarray analysis of human brain tissue from HIV+ patients with neuropathology or cognitive impairments have revealed alterations in similar genes and cellular processes to those observed in our study. Masliah et al. [*[*41*](https://www.ncbi.nlm.nih.gov/pmc/articles/PMC5197953/#B41-biomolecules-06-00043)*] reported down-regulation of genes involved in transcription, ion channels/transporters, cell-cycle molecules, and neuronal cytoskeleton components along with increased expression of interferon-stimulated genes in frontal cortex isolated from HIV encephalitis (HIVE) brains compared to HIV+ brains. Moreover, Gelman et al. [*[*42*](https://www.ncbi.nlm.nih.gov/pmc/articles/PMC5197953/#B42-biomolecules-06-00043)*] compared the gene expression in the neostriatum, neocortex, and white matter of patients with HIVE and neurocognitive impairment (NCI) to that of HIV-patients. Those results indicate strong similarities between the changes in gene expression in HIVE brains and those reported here for brains of CBA/SIV+ animals based on increased expression of interferon signaling, antigen presentation, and complement system genes. …..*

In this case, we select the full sentence with the citation marker as the context, as the sentence describes what was done in the cited study. The screenshot of the same annotation in brat is shown below.


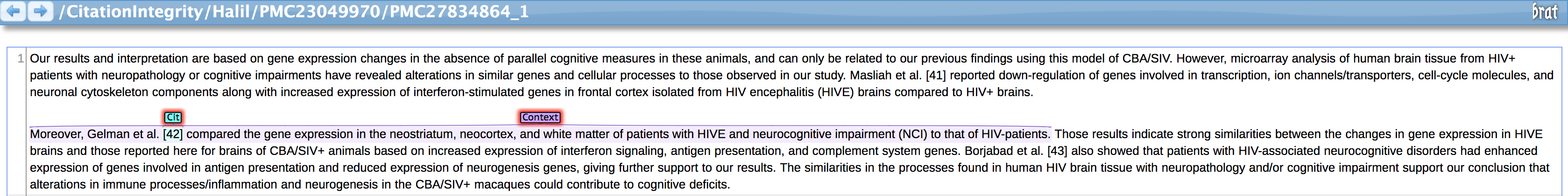


Citation 2:

*In summary, the results of this study show that CBA administration in SIV-infected macaques results in hippocampal up-regulation of genes involved in immune function and dysregulated expression of genes involved in neurogenesis. Similar patterns of gene expression are seen in brains of patients with HIVE and NCI [*[*42*](https://www.ncbi.nlm.nih.gov/pmc/articles/PMC5197953/#B42-biomolecules-06-00043)*], which we speculate suggests that over-activation of immune processes underlies the impairment of neurodevelopment. This is supported by the demonstrated capacity of inflammation to impair neurogenesis [*[*23*](https://www.ncbi.nlm.nih.gov/pmc/articles/PMC5197953/#B23-biomolecules-06-00043)*,*[*39*](https://www.ncbi.nlm.nih.gov/pmc/articles/PMC5197953/#B39-biomolecules-06-00043)*]. Thus, the results of this study support the hypothesis that inflammation-driven deficits in neurogenesis are a potential mechanism by which alcohol contributes to cognitive impairment in SIV-infected macaques. …..*

In this case, the context is a clause in the sentence, not the full sentence, as the rest of sentence includes a speculation by the authors of the citing paper. The context annotation in brat is shown below.


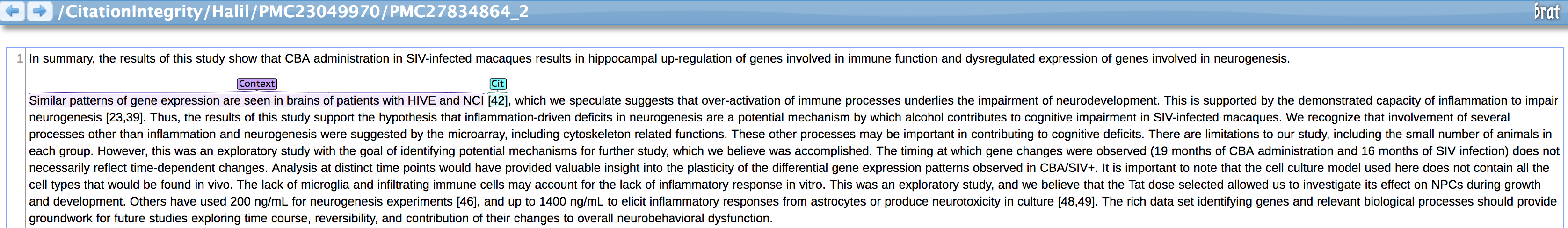


**Context annotation guidelines/caveats:**

- Include the full citation marker in the context annotation of the citation sentence. A citation marker can include one (e.g., [8]) or multiple references (e.g., [8-12]).
- Our goal is to capture what is being said in the citing paper about the reference article. Keep in mind that the citation context is ultimately used to identify relevant segments in the reference article.
- Multiple spans as citation context:
  - DO NOT annotate more than one sentence in a single Context object.
  - If annotating multiple clauses in a sentence for a given citation marker, annotate each one as a separate span rather than a single multi-part span.

*Sentence-level annotation is generally preferred.*

1. The whole sentence is generally best. For instance, the whole sentence can include context from other clauses or dependent clauses.
2. Do not make a fragment just to exclude phrases such as ‘X et al. (2019) suggest that’ from the context. Rather, annotate the entire sentence.
   1. *It is interesting to note that TREX1 has previously been implicated in the HIV-1 life cycle [42].*
   2. *Yan et al. proposed that TREX1 suppressed a type I IFN response that otherwise would be induced by HIV-1 infection [42].*
   3. *More recently, Lieberman and associates presented evidence that the cytosolic exonuclease TREX1 inhibits the innate immune response to HIV by degrading ssDNA derived from integrated provirus [36].*
   4. *It was demonstrated that in the absence of TREX1, a type I IFN response is induced that inhibits HIV replication and spreading, although the specific DNA sensor responsible remains to be identified [36].*

*Fragments of a sentence can be appropriate as the citation context span.*

1. *NOTE: If annotating a fragment, do not include ending punctuation (e.g., after the citation marker).*
2. Use a fragment to remove author’s speculations and inferences from citation context spans. These speculations and inferences would not be found in the reference paper.
   1. *Similar patterns of gene expression are seen in brains of patients with HIVE and NCI [*[*42*](https://www.ncbi.nlm.nih.gov/pmc/articles/PMC5197953/#B42-biomolecules-06-00043)*], which we speculate suggests that over-activation of immune processes underlies the impairment of neurodevelopment.*
   2. *Since claudin-low tumors possess features of stem cells [7], it is possible that the tumors that regress and recur or only partially regress are those that contain claudin-low cells.*
   3. EXCEPTION: In the following example, sentence-level annotation is preferable. It is not entirely clear whether ‘it might be possible’ is an inference from the reference paper or the author’s inference. Furthermore, ‘it might be possible’ may not be a substantial inference and the sentence resembles those in the sentence-level annotation (2) above. When in doubt, it is more acceptable to annotate the full sentence.
      1. *It might be possible that SAMHD1 acts in concert with another myeloid-specific co-factor [10].*
3. Use a fragment to remove author’s methods and results from citation context spans. These methods and results OF THE CITING PAPER would not be found in the reference paper.
   1. *We identified 10 predominantly IR–related SNPs by 1) their significant association with HOMA-IR (P < 0.05) in the MAGIC data (12), 2) significant association with fasting insulin in the MAGIC GWAS conditional on BMI or BMI-SNP interaction (28), and/or 3) evidence of association with IR-related traits such as lower HDL cholesterol, higher triglycerides, higher BMI, and higher waist-to-hip ratio (18).*
   2. *For example, although our studies assayed a similar number of postmortem cases, our sample consisted of all male autistic and control cases, while that of Voineagu et al. [20] consisted of 36% female autistic and 6% female control cases.*
   3. *Also, to identify gene expression abnormalities in the young autistic brain, we compared expression in the young autistic brain to the young control brain, while Voineagu et al. [20] lacked young controls.*
   4. *We also used available microarrays from the breast cancer cell lines [21], the UNC337-patient [20], the MERGE 550-patient dataset [47] and the NKI (295 patients [48], [49]).*
4. Use a fragment to remove text that is clearly not about the reference paper.
   1. *In addition, although it is well established that acute inhibition of mTOR impairs synaptic plasticity, it was recently reported that chronic treatment with rapamycin improved behavior of AD transgenic mice [13], [14], although effects were mild.*
   2. *This question is especially pertinent because interest in thermogenic drugs and drug targets has been rekindled by new evidence that brown adipose tissue can be active in adult humans and the discovery of new targets for drugs that might augment and activate brown adipose tissue (Fruhbeck et al., 2009; Wu et al., 2011; Bostrom et al., 2012; Fournier et al., 2012; Ye et al., 2012).*
5. Use a fragment if the segment can be cleanly split. For instance, you don’t need the entire sentence to understand what is referenced from the cited paper:
   1. *While TMAO could be derived from increased consumption of meat (Koeth et al., 2013), Prevotella has been previously associated with a dearth of meat in the diet (Wu et al., 2011).*
6. Do not fragment a sentence when there are multiple citations in the sentence but the full sentence context is useful to interpret the given citation. In the following example, it seems preferable to annotate the full sentence, instead of fragments (*‘dependent on PINK1 kinase activity’ OR* ‘*the autophagic elimination of damaged mitochondria’ and ‘is dependent on PINK1 kinase activity’)* here*.*
   1. *Upon mitochondrial damage or uncoupling, Parkin relocalizes to mitochondria and can mediate the autophagic elimination of damaged mitochondria (Narendra et al., 2008), which is dependent on PINK1 kinase activity (Geisler et al., 2010; Matsuda et al., 2010; Narendra et al., 2010; Vives-Bauza et al., 2010).*
7. The following sentence contains a parallel grammatical structure, which suggests that the full sentence should be annotated to capture the citation context.
   1. *Other randomized trials of epinephrine in hospitalized children have used as the control normal saline [22,25] , salbutamol [21] or both [24].*

*Occasionally, the citation context consists of multiple sentences.*

- Multiple sentences can be appropriate as context if the discussion of the reference paper extends beyond a single sentence.
  - Coreference (i.e., the use of pronouns, such as ‘they’, or noun phrases, such as ‘their findings’, to refer to the content of a reference paper discussed in a previous sentence) can indicate that multiple sentence annotation can be appropriate.
  - When annotating multiple sentences, annotate each sentence separately (i.e., do not extend your annotation to include multiple sentences).
- Continuation of the topic alone is not sufficient to take a preceding/following sentence, when what is being cited is clear and crisp from the sentence containing the citation marker alone.
  - *The stromal-vascular fraction (SVF) from fat depots of mice treated with EPO or saline was prepared and differentiated as described (20). At the beginning of differentiation induction, cells were treated with EPO, at 5 units/mL or at the dosage indicated, or with vehicle (PBS). 3T3-L1 and primary human adipocytes (H-adipocytes) were differentiated with EPO treatment for 9 days. SVF was differentiated to adipocytes with EPO treatment for 6 days.*
    - *In this example, while the sentences 2-4 seem to discuss the process in detail, they seem more related to the process used in the citing article, rather than the reference article, hence we only annotate the first sentence.*
- Inferences should not be added to the citation context. For example, one sentence is sufficient context in this case, the second sentence contains a further interpretation.
  - *Our findings are in agreement with the recent reports of Laguette et al. [20] and Hrecka et al. [19] that identified SAMHD1 as a Vpx and Vpx interacting protein. Taken together, these results demonstrate that Vpx targets SAMHD1 for ubiquitin-mediated degradation.*
- Information about different work should not be added. For example, author (Laguette et al.) is different from author [10] here:
  - *Indeed, Laguette et al. showed that over-expression of a HD domain mutant SAMHD1 in U937 cells fails to restrict HIV-1, suggesting that the phosphodiesterase activity of the HD domain is important for the restriction function of SAMHD1. Further analysis revealed that SAMHD1 blocks HIV-1 reverse transcription, as silencing SAMHD1 in THP-1 cells [9] and macrophages [10] increases the levels of viral DNA.*
- If the paragraph repeats the same citation multiple times, only connect a given instance with the closest Context span. Don't cross sentences.
  - EXAMPLE: 1st sentence [CITE]. 2nd sentence [CITE]*.* 3rd sentence [CITE].
    - In this case, cite within the sentence only, because there’s no need to go further in any sentence.
  - 1st sentence [CITE]. 2nd sentence. 3rd sentence [CITE].
  - 1st sentence [CITE]. 2nd sentence. 3rd sentence [CITE].
    - In this case, the 2^nd^ sentence could use the [CITE] in either the 1^st^ sentence or the 3^rd^ sentence but not both. (For the 1^st^ and 3^rd^ sentences, cite within, because there’s no need to go further in either.)

**Step 2. Evidence segment annotation and citation accuracy assessment:**

In this step, you are asked to annotate the reference article evidence segments relevant to the citation context, which forms the basis for citation accuracy verification. We ask that you identify up to five evidence segments (it can be fewer if the selected evidence segments are sufficient to make an accuracy assessment). A segment is typically a single sentence. However, in some cases, it may be impossible to identify single sentences as evidence segments, in which case you can label a section (a paragraph) or even the full article (see below on details about how to do this).

Since you’ll have read the reference articles already, we expect that finding these segments will be relatively straightforward^[[3]](#footnote-3)^. We expect that most citation contexts will refer to specific aspects of the reference article, so some sentences from the reference article must be annotated in most cases. Use the following priority order in creating evidence segments (i.e., an evidence segment in the title or abstract is more salient than one in the full-text).

- Title
- Abstract
- Full-text
  - Discussion/Conclusion
  - Results
  - Methods
  - Introduction

You will further classify each evidence segment for accuracy. When you highlight the evidence segment, you will also select from a drop-down list of ACCURATE and the major/minor error categories.

- If the citation is accurate, select the relevant segment in the reference article and label it as ACCURATE. The screenshot below shows three evidence segments that support the citation in the example above. The evidence segments are from the Abstract section of the reference article.


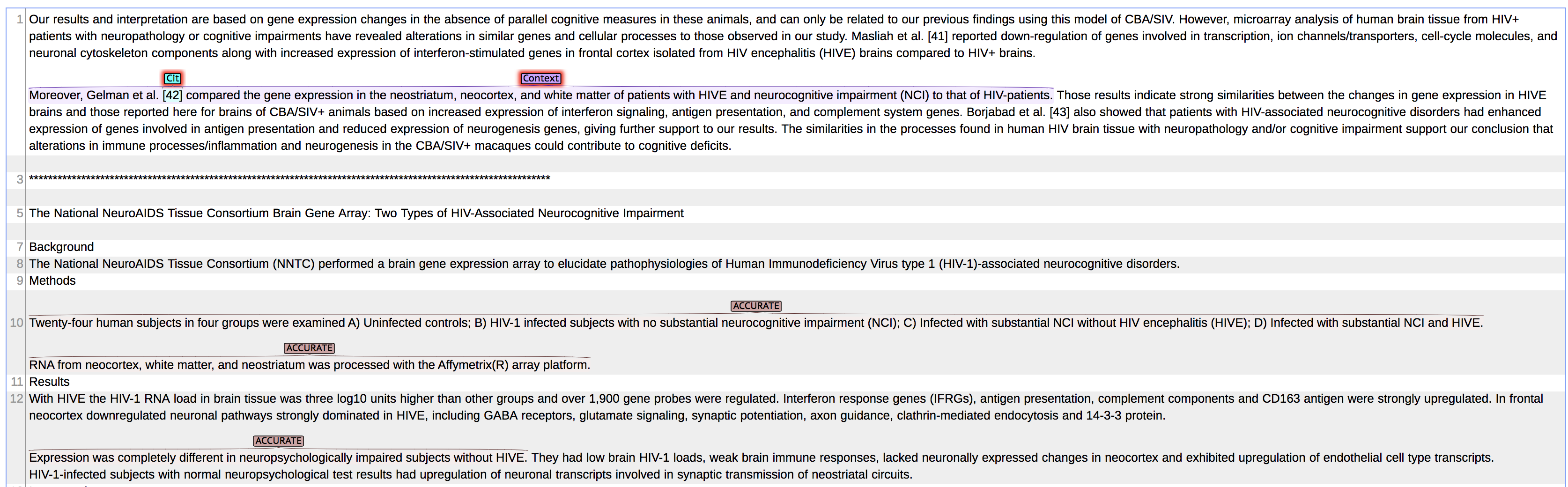


There are three major error categories and three minor citation error categories discussed in the literature. Major error categories are relevant when the citation context mischaracterizes the reference paper. Minor error categories are used when the citation context is not fully consistent with what is asserted in the reference paper but it does not have a major impact on the integrity of the citation. The major error types are as follows:

- CONTRADICT: the citation contradicts the assertion made in the reference article. In this case, the evidence segment is the segment in the reference article that contradicts the citation.
  - Example:
    - Citation: *“The average blood levels seen in our population (of human subjects) are below that usually associated with renal insufficiency.”*
    - Segment: *“in adult rats poisoned with lead when young, renal insufficiency persisted even after blood lead levels had fallen to normal”*
    - Explanation: The citation suggests that renal insufficiency is associated with higher than normal blood lead level, the reference article indicates that this is not the case.
- NOT_SUBSTANTIATE: the citation is relevant to the content of the reference article but the cited reference fails to substantiate the assertion made in the citing paper. In this case, the evidence segment is the segment where the original assertion is made. Another case is when the citation context contains multiple claims but not all claims are substantiated.
  - Example:
    - Citation: “*Benefits of percutaneous screw fixation are a quicker time to union and a more rapid return to sport and work”*
    - Segment: “*Patients treated with a cast returned to work at a mean of six weeks, while those managed with an operation returned to work at a mean of five weeks (p = 0.381), and both groups were able to perform most work tasks comfortably within two weeks of return.”*
    - Explanation: The quoted study did not assess time to union or return to sport, and showed no difference in time to return to work. A possible reason for this common type of inaccuracy is that authors often try to substantiate important statements with as much literature as possible. The articles are often merely sampled and not read thoroughly.
- IRRELEVANT: the citation is irrelevant to the content of the reference article. There is no information in the reference article that substantiates the citations.
  - Example:
    - Citation: *“increased risk of esophageal cancer with alcohol consumption.”*
    - Explanation: the cited article is about treatment and makes no mention of cancer etiology.

The minor error types are the following:

- MISQUOTE: The numbers or percentages are misquoted.
  - Example:
    - Citation: “42 patients”
    - Segment: “42 abscesses in 40 patients”
- OVERSIMPLIFY: The findings of the reference article are oversimplified or overgeneralized.
  - Example:
    - Citation: *“The established treatment for undisplaced scaphoid fractures is immobilisation in a cast for 8-12 weeks.”*
    - Segment: *“Fractures of the middle or distal thirds should be immobilised in an unpadded plaster cast for six weeks and fractures of the proximal third and all vertical oblique fractures should be immobilised for ten to twelve weeks.”*
    - Explanation: In this example the distinction is lost because of the summary.
- INDIRECT (relevant when the reference article is a review article): A citation is made to the review article, whereas it would be more appropriate to cite the research article discussed in the review article.
- ETIQUETTE: The citation is in an ambiguous location.
  - Example:
    - Citation: There is now evidence that HIV-1 has the potential to stimulate the innate immune system through its RNA, DNA, and capsid [16], [34], **[36]**, [37].
    - Segment: *“Therefore our working model of the innate immune pathway activated by cytosolic HIV DNA starts with an unknown sensor (that may preferentially recognize ssDNA) that signals through STING, TBK1 and IRF3 to activate IFN expression”*
    - Explanation: The paper discusses RNA and DNA, but not capsid. In this case, it seems that the cited papers discuss at least one of RNA, DNA, or capsid, but not all. A more appropriate citation could be like: *..its RNA [15], [34], DNA, [36], and capsid [37].*

NOTE: If you can identify a citation as an error but, in your opinion, it does not fall into any of the sub-categories above, you can annotate Major or Minor as the category. In these cases, use the Note field to add a short note about the type of error you observe. The Note field is free text. Be consistent in your notes (use the same note if you see multiple cases of the same error). This can help us determine the potential shortcomings of the categorization above.

The dialog box from which the category is selected is shown in the screenshot below.


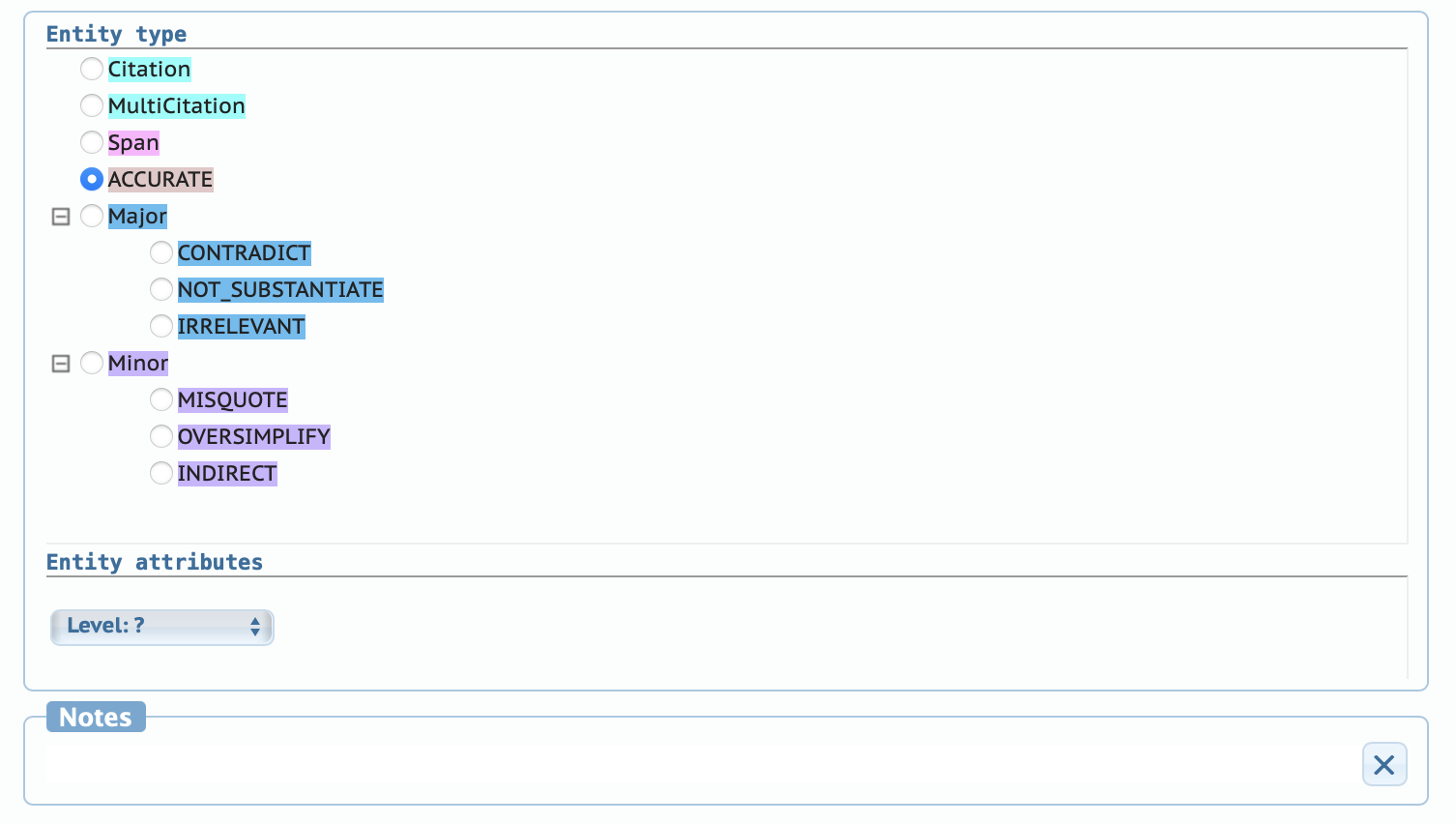


Annotating sentences vs. sections vs. full article as segments:

We expect that most evidence segments annotations will be made at the sentence level. This is the default setting.

For some citations, it may be impossible to select particular sentences, especially when the citation is somewhat vague or refers to the reference article without much specificity. In these cases, you can use Section level or Article level annotations.

- Section allows you to identify a paragraph as the relevant evidence segment. In this case, highlight the first word of the relevant section for annotation, select the category, and select Level = ‘Section’ from the “Level=?” drop-down shown at the bottom of the screenshot above. Note that when you use the Section level, we will assume that all sentences in the section can be included in the evidence segment.
- Article should be used in limited cases. For example, the citation may be overly broad (e.g., the reference paper reports a particular dataset and the citation is simply the dataset name). Or, there is no relevant information in the article (so, IRRELEVANT is the category to pick). In these cases, select the title of the reference article as the segment and use Level=’Article’.

**Evidence segment annotation guidelines/caveats:**

- The unit of annotation for evidence segments is the sentence. Do not annotate spans shorter than a sentence (the title is considered a sentence). If consecutive sentences form evidence segments, annotate them as multiple evidence segments (unless Section level annotation is used, i.e., the first word of the paragraph/section is annotated).
- You can annotate fewer than five sentences.
- According to the literature, multi-citations are likely to have accuracy errors, so play special attention to these.
- The relevant information for the citation can be in a table or a figure in the reference article. In such cases, annotate as the evidence segment the sentence(s) that refer to the table or figure in the reference article text (e.g., “The results are shown in Table 1.”). If there is no reference to the table or figure, you can annotate the table or figure caption.
- Sometimes, the reference is to information in the supplementary files of the reference article. You are not expected to identify such information. Make your judgement based on the text available to you in brat.
- When making error vs. accurate judgements, try to limit your inferencing and over-interpretation. You do not need to give benefit of doubt to the authors.
- Sentence annotations are preferable to Section annotations, which are preferable to Article annotations (the order or preference: Sentence > Section > Article).
- In the Level=’?’ drop-down, there is also Level=’Sentence’, which is the default setting, so it doesn’t need to be selected.
- To the extent possible, avoid using Section level if most relevant sentences within the section can be identified and not all sentences are relevant.
- Avoid Abstract section as a Section level annotation (in other words, selecting ’Abstract’ as the evidence segment and labeling the level as ‘Section’). This essentially means that the entire abstract is referenced in the citing paper, which itself is not useful for us.
- If no relevant information to the citation is found, annotate the title as Level=’Article’ with the label IRRELEVANT.
- Authors often hedge when citing to ensure that the articles they are citing seem supportive, so make sure that when they cite scientific claims from a reference article, these claims are actually made in the reference article. Otherwise, use NOT_SUBSTANTIATE.
- **You may annotate more than one evidence segment if there are segments that relate to the citation in different ways, but only annotate one segment type (e.g., ACCURATE, OVERSIMPLIFY) for a single context annotation.**
  - If there is more than one segment type, select the higher type in the hierarchy below.
  - Hierarchy:
    - MAJOR
      - CONTRADICT
      - IRRELEVANT
      - NOT_SUBSTANTIATE
    - MINOR
      - MISQUOTE
      - OVERSIMPLIFY
      - INDIRECT
      - ETIQUETTE
    - ACCURATE

**Brat tips/quirks:**

- It is easy to miss a character or annotate an additional character, when selecting long spans for annotation with brat. After you add your annotation, make sure to check that the span is captured correctly, by hovering over the label.
- When two annotations, say a Citation and a Context annotation that indicates the context, overlap, brat puts a red border around their labels, indicating this might be an error. Ignore these errors.
- Alert us if you see warning/error messages in red when you open a brat document.
- You may see other strange behavior (like text becoming impossible to read) when long text spans are accidentally selected. Alert us in these cases, because we need to fix your annotation file.

**Appendix.**

Some error examples are listed below. More papers discussing error examples are in the Box folder: <https://uofi.box.com/s/tszudq1zogvylsxk51uasmo6pp90dm3n>.

**Error examples**

From Mogull (2017): Accuracy of cited “facts” in medical research articles: A review of study methodology and recalculation of quotation error rate. PLoS One. 2017 Sep 14;12(9):e0184727.


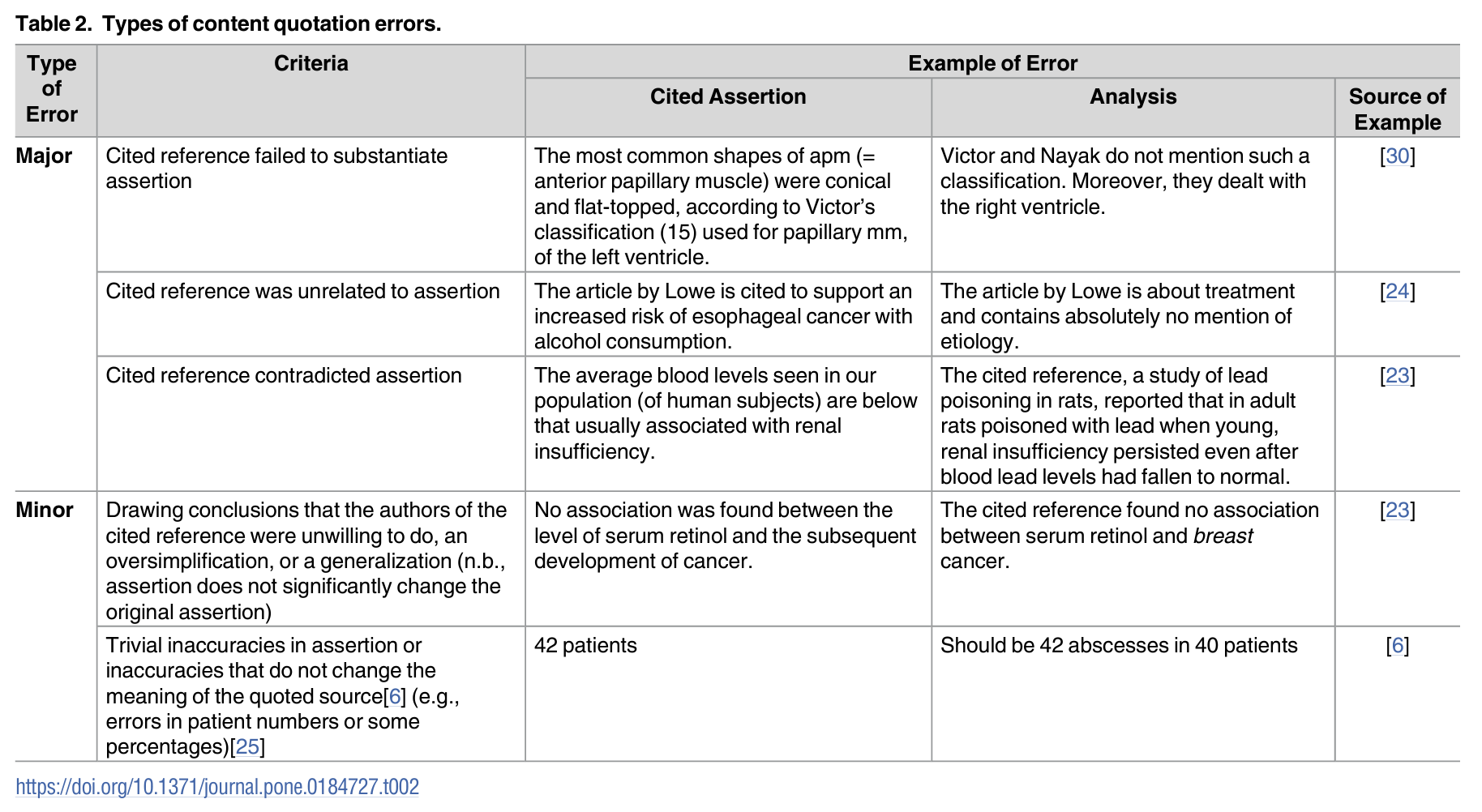


From Buijze et al. (2012): Predictors of the accuracy of quotation of references in peer-reviewed orthopaedic literature in relation to publications on the scaphoid. The Journal of Bone and Joint Surgery. British volume. 2012;94(2):276-80.

1. Oversimplification:

*The patient should be treated as a soft tissue injury of the wrist and followed up appropriately.*

This was quoted in support of

*Equivocity of conventional radiographs often dictate a defensive strategy with cast immobilisation for at least 10 days.*

The statement is only partially supported, as many patients in the study had fewer than ten days of immobilisation and the authors did not feel that was suboptimal.

From Tfelt-Hansen (2015): The qualitative problem of major quotation errors, as illustrated by 10 different examples in the headache literature. Headache: The Journal of Head and Face Pain. 2015 Mar;55(3):419-26. (more examples in this article)

1. Overgeneralization:

In a debate on a possible vascular mechanism of migraine, an article by Nagata et al from 2009 was quoted as follows: “Nagata et al reported that spontaneous migraine was not associated with any dilation of the middle meningeal artery as measured by MRA.” However, readers of the quoted article will note already from the title, “The middle meningeal artery during a migraine attack: 3T magnetic resonance angiography study,” that only 1 migraine attack was studied.

1. Fail to substantiate:

A pharmacokinetic study of a newly developed administration form of orally inhaled dihydroergotamine (DHE) from 2008 quoted a previous review article on DHE13 as follows: “Other routes of administration such as nasal delivery (40% bioavailability) have erratic and somewhat unpredictable pharmacokinetic (PK) properties that pose therapeutic challenges (e.g., unpredictable clinical response or adverse events).” Yet the referenced review article contains no mention at all about supposedly inconsistent kinetics of nasal DHE.

1. Fail to substantiate:

This quotation is from a review of drug therapy for migraine published in the New England Journal of Medicine in 1993, the year in which Imitrex (sumatriptan) was introduced in the United States: “Oral sumatriptan was more effective in relieving headache than either ergotamine plus caffeine or aspirin plus metoclopramide, but the rate of recurrence was higher with sumatriptan.” In 1 of the quoted articles, it was shown that oral sumatriptan 100 mg was superior to Cafergot (2 mg ergotamine tartrate plus 200 mg caffeine), producing more headache relief (HR) (66% vs 48%; P < 0.001), but with more recurrences within 48 hours (41% vs 30%; P < 0.01). In the other study, sumatriptan 100 mg was not significantly superior to a combination of 900 mg aspirin and 10 mg metoclopramide for the first treated attack (HR 56% vs 45%; P = 0.078), the primary efficacy parameter, but was superior for the second (HR 58% vs 34%; P = 0.001), and third (HR 65% vs 34%; P < 0.001) treated attacks and for some other parameters. The important point is that there was no difference between sumatriptan and aspirin plus metoclopramide regarding the primary efficacy parameter; thus, one cannot use this study to claim that sumatriptan is superior to aspirin plus metoclopramide.

1. Experimental Settings

**Citation context identification:** To deal with class imbalance (i.e., most sentences are non-context sentences), we used focal loss [1], a modification of standard cross-entropy loss. The following hyperparameters were used: batch size=32, dropout=0.2, optimizer=AdamW [2]. For the focal loss, we set γ= 2.0 and α= 0.25.

**Evidence sentence retrieval:**

We use the *monot5-base-med-msmarco* version [3] which consists of a T5-base reranker that was fine tuned on MS MARCO passage dataset [4] for 10k steps and further fine-tuned on a medical subset of MS MARCO [5] for 1k steps.

**Citation accuracy classification using MultiVerS:**

We used the default hyperparameters of the MultiVerS model. Specifically, the maximum sequence length is set to 4096, with a hidden size of 1024, a learning rate of 5e-05, a batch size of 1. We trained the models for a total of 5 epochs.

[1] Lin TY, Goyal P, Girshick R, He K, Dollár P. Focal loss for dense object detection. InProceedings of the IEEE international conference on computer vision 2017 (pp. 2980-2988).

[2] Zhang Z. Improved Adam optimizer for deep neural networks. In2018 IEEE/ACM 26th international symposium on quality of service (IWQoS) 2018 (pp. 1-2). IEEE.

[3] Pradeep R, Ma X, Nogueira R, Lin J. Scientific Claim Verification with VerT5erini. In Proceedings of the 12th International Workshop on Health Text Mining and Information Analysis 2021 Apr (pp. 94-103).

[4] Bajaj P, Campos D, Craswell N, Deng L, Gao J, Liu X, Majumder R, McNamara A, Mitra B, Nguyen T, Rosenberg M. MS MARCO: A human generated machine reading comprehension dataset. arXiv preprint arXiv:1611.09268. 2016 Nov 28.

[5] MacAvaney S, Cohan A, Goharian N. SLEDGE: a simple yet effective baseline for COVID-19 scientific knowledge search. arXiv preprint arXiv:2005.02365. 2020 May 5.

# Prompt Structure


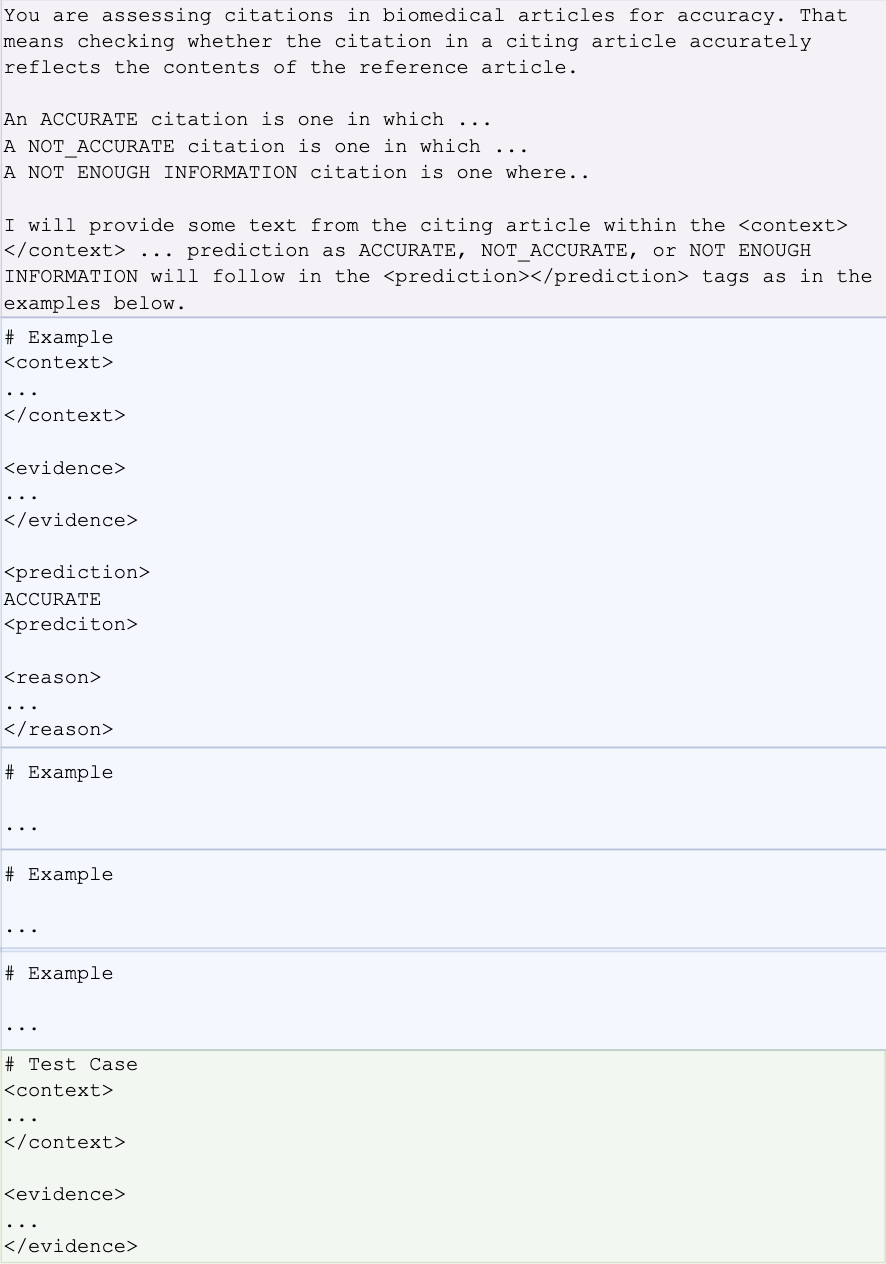


**Example Prompt:**

You are assessing citations in biomedical articles for accuracy. That means checking whether the citation in a citing article accurately reflects the contents of the reference article.

An ACCURATE citation is one in which the information provided aligns well with facts, evidence, and established knowledge. The exact details should match explicitly and not just the general theme for it to be ACCURATE.

A NOT_ACCURATE citation is one in which the citation context contradicts the passage from the reference article, or does not substantiate it, or oversimplifies it, or distorts its meaning, or misquotes it.

A NOT ENOUGH INFORMATION citation is one where the reference article is irrelevant to the citation i.e. the citation content refers to some information that is not explicitly discussed in the evidence.

I will provide some text from the citing article within the <context></context> tags and highlight the citation marker within that context as <|cit|> (single citation) or <|multi_cit|> (multi-citation). There might be other citations in the same context which I will highlight as <|other_cit|> that are not relevant to your assessment. Then I will provide a few sentences from the reference article as evidence within <evidence></evidence> tags. Each sentence in evidence will be marked indexed by a number in parentheses. The prediction as ACCURATE, NOT_ACCURATE, or NOT ENOUGH INFORMATION will follow in the <prediction></prediction> tags as in the examples below.

**# Example**

***<context>***

Disease related studies that involve microbiome analysis using metagenomics include obesity [<|other_cit|>], Crohn’s disease [<|multi_cit|>], type 2 diabetes [<|other_cit|>] and many others [<|other_cit|>].

***</context>***

***<evidence>***

[0] This study's inferred IBD metagenomes include broadly increased oxidative metabolism, decreased SCFA production, and increased mucin degradation relative to healthy subjects. [1] The microbiome of ileal Crohn's disease was notable for increases in virulence and secretion pathways. [2] An important consideration that informed the remainder of our analysis, and which is often overlooked in studies of the microbiome, was the consistent covariation among disease status, aspects of subject environment, and microbiome structure. [3] We have thus analyzed the GI microbiomes of 121 CD patients, 75 UC patients, and 27 healthy controls using a novel multivariate metagenomic analysis pipeline specifically accounting for environmental factors (including treatment, age, and tobacco use). [4] In addition to assessing microbiome composition, we have analyzed the inferred metagenome as determined from phylogenetically-associated reference genomes, including metabolic modules and pathways also associated with disease status and with environmental factors such as medications and smoking.

***</evidence>***

***<prediction>***

ACCURATE

***</prediction>***

***<reason>***

The context states that studies involving microbiome analysis using metagenomics include Crohn's disease. The evidence sentences support this by mentioning:

Analyzing the microbiome of Crohn's disease patients (sentences 1, 3)

Finding changes in metabolic pathways and microbiome composition associated with Crohn's disease status (sentences 2, 4)

Therefore, the citation accurately reflects that metagenomic studies have analyzed the microbiome in Crohn's disease. The evidence aligns with and substantiates the information provided in the citing context.

Since the claiming text's statement about Crohn's disease metagenomic studies is corroborated by the details in the evidence, I assess this citation as accurate. The citation context and evidence are in agreement.

***</reason>***

**# Example**

***<context>***

This is coherent with the fact that hACE2 expression were not observed in the gut of the mice used in that study [<|cit|>].

**</context>**

***<evidence>***

[0] Although hACE2 is expressed in the gastrointestinal tract in other hACE2-expressing mice, productive infection was observed only upon intragastric inoculation or at early time points following intranasal infection11,12. [1] In the gastrointestinal tract of K18-hACE2 mice, hACE2 was expressed most abundantly in the colon, which correlated with infection seen at later time points. [3] The spleen in SARS- CoV-2-infected K18-hACE2 mice appeared normal (Extended Data Fig 2f), and fibrin thrombi were not detected in any of the extra-pulmonary organs examined. [4] We saw substantial immune cell accumulation in the lungs of K18-hACE2 mice, an observation consistent with post-mortem analysis of human patients39. [5] mRNA expression levels of hACE2 in the lung, kidney, heart, brain, spleen, duodenum, colon, and ileum of naive K18 hACE2 mice (two experiments, n = 5).

***</evidence>***

***<prediction>***

NOT_ACCURATE

***</prediction>***

***<reason>***

The context states that hACE2 expression were not observed in the gut. But sentence [1] in the evidence states that hACE2 was expressed most abundantly in the colon. Since the colon is a part of the gut, the citation context contradicts the evidence and hence the citation is NOT_ACCURATE.

***</reason>***

**# Example**

***<context>***

All analyses were performed using R software version 3.0.2 (library survival) and Stata 13.0 (College Station, TX) [<|cit|>].

***</context>***

***<evidence>***

[0] ### Statistical Analyses [1] Sensitivity analyses: Restricting to cohorts with death from registries. [2] To further investigate this issue, we conducted sensitivity analyses taking into account loss to follow-up, and were reassured to find little differences in our estimates. [3] We calculated all-cause mortality rates (per 1,000 person-years) by dividing the total number of deaths by the total number of person-years of observation. [4] In effort to reduce bias from informative censoring, we used weighted regression methods to estimate mortality rates, using an indicator variable that was created to identify participants who were lost to follow-up.

***</evidence>***

***<prediction>***

NOT ENOUGH INFORMATION

***</prediction>***

***<reason>***

R or Stata was not mentioned in the evidence segment and there is no mention of the software used in the evidence segment. The evidence is not sufficient to decide if R and Strata were used and hence the citation is NOT ENOUGH INFORMATION.

***</reason>***

**# Example**

***<context>***

We have previously shown an even higher mortality rate, around 30 deaths per 100 person-years, during the pre-treatment screening period [<|other_cit|>] in this population and recent reviews show 32% loss to follow-up during this pre-treatment period despite eligibility for ART [<|cit|>], a 20% loss to follow-up among patients within the first year of antiretroviral therapy [<|other_cit|>], among whom mortality is very high [<|other_cit|>].

***</context>***

***<evidence>***

[0] While pre-ART loss to care may not pose as immediate a mortality threat as loss of patients who already have clinical AIDS, it is still a major impediment to improving the outcomes of HIV care and treatment overall, is itself a contributor to the high mortality observed during the first year on ART, and wastes scarce health system resources. [1] Studies of retention in pre-ART care report substantial loss of patients at every step, starting with patients who do not return for their initial CD4 count results and ending with those who do not initiate ART despite eligibility. [2] Difficult as this problem is for managing ART patients, it is even worse during the pre-ART period, because patients are expected to visit the clinic less frequently, and more clinics are able to provide pre-ART services than are accredited to offer ART. [3] In particular, the estimate of the overall loss of patients during pre-ART care is likely to be imprecise. [4] The rate of early mortality and loss to follow-up, which itself portends mortality for many, averages 23% across the region.

***</evidence>***

***<prediction>***

NOT_ACCURATE

***</prediction>***

***<reason>***

Sentence [4] states an average loss of 23% across the region which is misquoted as 32% in the citation context. Since, the numbers or percentages do not match, the citation is NOT_ACCURATE.

***</reason>***

**# Test Case**

***<context>***

Over the past 4 years, FMO3 and TMAO have emerged as key components of a complex axis integrating diet and the gut microbiome with atherosclerosis<|multi_cit|>, and knockdown of FMO3 was recently shown in mouse models of hyperlipidemia to prevent atherosclerosis and improve the metabolic phenotype<|other_cit|>.

***</context>***

***<evidence>***

[0] These results are consistent with a gut microbiota dependent mechanism whereby generation of TMAO impairs RCT, potentially contributing to the observed pro-atherosclerotic phenotype of these interventions. [1] These findings raise the possibility that other dietary nutrients possessing a trimethylamine structure may also generate TMAO from gut microbiota and promote accelerated atherosclerosis. [2] Using isotope tracer studies, clinical studies, and animal models employing germ-free mice, we demonstrate a role for gut microbiota metabolism of L-carnitine in atherosclerosis pathogenesis. [3] Conversely, an increased synthetic capacity for microbiota-dependent production of TMAO from carnitine may contribute to atherosclerosis, particularly in omnivores where carnitine consumption is elevated. [4] (c) Summary scheme outlining pathway for microbiota participation in atherosclerosis via metabolism of dietary carnitine and choline forming TMA and TMAO, as well as the impact of TMAO on cholesterol and sterol metabolism in macrophages, liver and intestines.

***</evidence>***

# Full citation accuracy classification results

Table S2. Citation accuracy classification results. Top-k sentences are retrieved using BM25 + MonoT5 reranker. In-context learning uses top 5 sentences only.

| Evidence Input | Label | Precision | Recall | F1 | Micro-F1 | Macro-F1 |
| --- | --- | --- | --- | --- | --- | --- |
| *MultiVerS models* | | | | | | |
| Oracle (gold evidence sentence) | ACCURATE | 0.79 | 0.80 | 0.79 | 0.73 | 0.75 |
|  | NOT_ACCURATE | 0.52 | 0.51 | 0.52 |  |  |
|  | IRRELEVANT | 0.91 | 0.96 | 0.93 |  |  |
| Title + abstract | ACCURATE | 0.72 | 0.67 | 0.69 | 0.56 | 0.43 |
|  | NOT_ACCURATE | 0.36 | 0.41 | 0.38 |  |  |
|  | IRRELEVANT | 0.19 | 0.22 | 0.20 |  |  |
| Top 5 sentences | ACCURATE | 0.74 | 0.65 | 0.69 | 0.58 | 0.50 |
|  | NOT_ACCURATE | 0.39 | 0.48 | **0.43** |  |  |
|  | IRRELEVANT | 0.34 | 0.40 | 0.37 |  |  |
| Top 10 sentences | ACCURATE | 0.72 | 0.62 | 0.67 | 0.56 | 0.48 |
|  | NOT_ACCURATE | 0.36 | 0.47 | 0.41 |  |  |
|  | IRRELEVANT | 0.34 | 0.38 | 0.36 |  |  |
| Top 20 sentences | ACCURATE | **0.76** | 0.64 | 0.69 | 0.59 | **0.52** |
|  | NOT_ACCURATE | 0.38 | **0.50** | **0.43** |  |  |
|  | IRRELEVANT | **0.41** | 0.44 | 0.42 |  |  |
| Top 20 sentences + annotated evidence | ACCURATE | 0.74 | 0.65 | 0.69 | 0.58 | 0.50 |
|  | NOT_ACCURATE | 0.39 | 0.49 | **0.43** |  |  |
|  | IRRELEVANT | 0.37 | 0.38 | 0.38 |  |  |
| *In-context learning* | | | | | | |
| GPT-3.5-turbo | ACCURATE | 0.68 | 0.79 | 0.73 | 0.57 | 0.38 |
|  | NOT_ACCURATE | 0.26 | 0.03 | 0.05 |  |  |
|  | IRRELEVANT | 0.23 | 0.64 | 0.34 |  |  |
| GPT-4 | ACCURATE | 0.71 | **0.90** | **0.80** | **0.65** | 0.45 |
|  | NOT_ACCURATE | **0.66** | 0.05 | 0.09 |  |  |
|  | IRRELEVANT | 0.35 | **0.74** | **0.48** |  |  |

# Error Analysis

For the best-performing MultiVerS model (citance as citation context and top-20 sentences from BM25 + MonoT5 reranker for evidence sentence retrieval), we provide the confusion matrix as well as the translation of 3-way classification to the fine-grained error categories used in annotation in Table S3. It shows that IRRELEVANT citations are often mistaken for NOT_ACCURATE citations (42%), while NOT_ACCURATE citations are often confused as ACCURATE citations (43%). There is also significant confusion of ACCURATE citations as NOT_ACCURATE (30%). Within the major error categories, the model exhibited the highest performance in predicting instances labelled as NOT_SUBSTANTIATE (0.52 F1). Among the minor error categories, the model demonstrated reasonable performance in predicting the MISQUOTE category (0.67 F1). Among error categories, CONTRADICT and OVERSIMPLIFY are most challenging and tend to be predicted more often as ACCURATE.

Table S3. Confusion matrix for the best-performing MultiVerS model. Rows represent true classes, and columns represent predicted labels. The bottom part of the table shows the mapping of 3-way class predictions to fine-grained labels used in annotation.

|  | ACCURATE | NOT_ACCURATE | IRRELEVANT |
| --- | --- | --- | --- |
| ACCURATE | 0.65 | 0.30 | 0.05 |
| IRRELEVANT | 0.14 | 0.42 | 0.44 |
| NOT_ACCURATE | 0.43 | 0.50 | 0.07 |
| *Fine-grained error categories* | | | |
| INDIRECT | **0.56** | 0.38 | 0.06 |
| ETIQUETTE | 0.38 | **0.58** | 0.04 |
| OVERSIMPLIFY | 0.53 | **0.40** | 0.06 |
| NOT_SUBSTANTIATE | 0.31 | **0.52** | 0.17 |
| CONTRADICT | 0.67 | **0.25** | 0.08 |
| MISQUOTE | 0.33 | **0.67** | 0.00 |

We also examined instances where all models failed to identify the correct label (94 cases). We categorize the most common errors into the following categories to shed light on future work to improve the citation accuracy model's performance. It was difficult to determine the reason for misclassification in other cases.

- **Citation contexts with multiple citations** (23 cases). Other ciations in the context might have biased the predictions. In the example below, partial information like ``the G allele had been previously associated with an increased risk of type 1 diabetes'' came from different sources.

**Citation context:** Interestingly, the G allele had been previously associated with an increased risk of type 1 diabetes (11) but a lower risk of T2D (10). (PMC3749360)

- **Subtle differences (e.g., differences in numbers) are not recognized by the model.** (16 cases). The sentence retriever can capture sentences quite similar to the citation context, yet the differences are subtle and difficult to discern for the model In the example below, the citation context indicates “63%'” which fails to properly denote the decimals as presented in the reference article.

**Citation context:** Subsequent models using genome-wide complex trait analysis suggested that 63% of T2D susceptibility might be attributable to common genetic variation in the full set of GWAS SNPs (12). (PMC4030114)

- **Citation broadly refers to the article but not to its content** (6 cases). In future work, a classifier can be developed to assess whether a citation *needs* to be checked for accuracy.

**Citation context:** The eighth most cited article was by Butler and Barrientos [10] and published in Brain Behavior and Immunity. (PMC9186477)

- **Citation context with additional information irrelevant to the citation** (5 cases). Citation context includes additional information that is not relevant to the reference article. A fine-grained, span-level citation context formulation could improve the predictions in such cases.

**Citation context:** Interestingly, we observed approximately 2 kg weight loss among obese individuals in our study, whereas Flanagan et al. (16) found that the percentage of obese individuals who had gained weight during the lockdown was higher than that of those with normal weight (33 vs. 27%) (16). (PMC7675243)

- **The citation context is incomplete due to sentence splitter issues** (5 cases).

**Citation context:** 83–90) provides important insights in the complex interplay between asthma, ICS, SARS-CoV-2 infection and COVID-19 (8). (PMC7328313)

- **Lack of sufficient context in the citance** (3 cases). For example, lack of coreference information from the surrounding citing context may result in insufficient details for the retrieval model to find corresponding information from the reference article for verification.

**Citation context:**  Such a discrepancy between Flanagan et al. study (16) and ours may be attributed to the time frame of the data collection. (PMC8801912)

We randomly selected 20 error examples and expanded the citation context with desired information or removed unnecessary context information. Then, we used the new citation context to retrieve new evidence sentences from reference articles and tested citation accuracy classification. Citation context adjustment led to 4 correct predictions (out of 20), which suggests that refinements on citation context identification could improve results further.

# GPT-3.5-turbo experiments

As additional experiments, we fine-tuned GPT-3.5 model (*gpt-3.5-turbo-0613*) with 100 examples for each class sampled randomly from the training set, which showed a marginal improvement in the performance for the NOT_ACCURATE class but a regression in performance of the other classes leading to lower overall performance, as shown in Table S4. Similar results were observed when the problem was posed as a binary classification problem (by merging the IRRELEVANT class into the NOT_ACCURATE class) with a low recall of 0.2 for the NOT_ACCURATE class.

Table S4. Assessing the impact of fine-tuning and a simplified task formulation on GPT-3.5-turbo model. For fine-tuning, the training set contained 100 samples each for all the three classes. When posing as binary classification, the IRRELEVANT class was merged into the NOT_ACCURATE class.

| **GPT-3.5 setting** | **Label** | **Precision** | **Recall** | **F1** | **Micro-F1** | **Macro-F1** |
| --- | --- | --- | --- | --- | --- | --- |
| Original | ACCURATE | 0.68 | 0.79 | 0.73 | 0.57 | 0.38 |
|  | NOT_ACCURATE | 0.26 | 0.03 | 0.05 |  |  |
|  | IRRELEVANT | 0.23 | 0.64 | 0.34 |  |  |
| Fine-tuning | ACCURATE | 0.74 | 0.56 | 0.64 | 0.47 | 0.37 |
|  | NOT_ACCURATE | 0.39 | 0.11 | 0.17 |  |  |
|  | IRRELEVANT | 0.18 | 0.94 | 0.30 |  |  |
| Binary task formulation | ACCURATE | 0.66 | 0.91 | 0.77 | 0.65 | 0.53 |
|  | NOT_ACCURATE | 0.55 | 0.20 | 0.30 |  |  |

1. For more information about brat, including tutorials: <https://brat.nlplab.org/>. [↑](#footnote-ref-1)
2. Brat annotation tool removes all the formatting, so these IDs might be useful in locating the articles on PubMed Central (PMC) to see its structure more clearly. Typing the ID into Google Search or PMC search engine (<https://www.ncbi.nlm.nih.gov/pmc/>) will retrieve the article. [↑](#footnote-ref-2)
3. We make the reference articles available as PDF files in the Box folder. [↑](#footnote-ref-3)
